# Efficient gene orthology inference via large-scale rearrangements

**DOI:** 10.1186/s13015-023-00238-y

**Published:** 2023-09-28

**Authors:** Diego P. Rubert, Marília D. V. Braga

**Affiliations:** 1 Faculdade de Computação, Universidade Federal de Mato Grosso do Sul, Campo Grande, Brazil; 2 Faculty of Technology and Center for Biotechnology (CeBiTec), Bielefeld University, Bielefeld, Germany

**Keywords:** Comparative genomics, Double-cut-and-join, Indels, Gene orthology

## Abstract

**Background:**

Recently we developed a gene orthology inference tool based on genome rearrangements (*Journal of Bioinformatics and Computational Biology* 19:6, 2021). Given a set of genomes our method first computes all pairwise gene similarities. Then it runs pairwise ILP comparisons to compute optimal gene matchings, which minimize, by taking the similarities into account, the weighted rearrangement distance between the analyzed genomes (a problem that is NP-hard). The gene matchings are then integrated into gene families in the final step. The mentioned ILP includes an optimal *capping* that connects each end of a linear segment of one genome to an end of a linear segment in the other genome, producing an exponential increase of the search space.

**Results:**

In this work, we design and implement a heuristic capping algorithm that replaces the optimal capping by clustering (based on their gene content intersections) the linear segments into $m\ge 1$ subsets, whose ends are capped independently. Furthermore, in each subset, instead of allowing all possible connections, we let only the ends of content-related segments be connected. Although there is no guarantee that *m* is much bigger than one, and with the possible side effect of resulting in sub-optimal instead of optimal gene matchings, the heuristic works very well in practice, from both the speed performance and the quality of computed solutions. Our experiments on primate and fruit fly genomes show two positive results. First, for complete assemblies of five primates the version with heuristic capping reports orthologies that are very similar to the orthologies computed by the version of our tool with optimal capping. Second, we were able to efficiently analyze fruit fly genomes with incomplete assemblies distributed in hundreds or even thousands of contigs, obtaining gene families that are very similar to ${\text{F}} {\textsc{ly}} {\text{B}} {\textsc{ase}}$ families. Indeed, our tool inferred a higher number of complete cliques, with a higher intersection with ${\text{F}} {\textsc{ly}} {\text{B}} {\textsc{ase}}$, when compared to gene families computed by other inference tools. We added a post-processing for refining, with the aid of the ${\textsc{mcl}}$ algorithm, our *ambiguous* families (those with more than one gene per genome), improving even more the accuracy of our results. Our approach is implemented into a pipeline incorporating the pre-computation of gene similarities and the post-processing refinement of ambiguous families with $\textsc {mcl}$. Both the original version with optimal capping and the new modified version with heuristic capping can be downloaded, together with their detailed documentations, at https://gitlab.ub.uni-bielefeld.de/gi/FFGC or as a Conda package at https://anaconda.org/bioconda/ffgc.

**Supplementary Information:**

The online version contains supplementary material available at 10.1186/s13015-023-00238-y.

## Background

The study of distances and parsimonious evolutionary scenarios based on large-scale genome rearrangements traditionally depends on the pre-computation of *gene families*. Computing such a distance is usually polynomial when genomes have at most one gene per family [1–3] or NP-hard otherwise [4–8]. These works adopt several rearrangement models and among the most popular ones is the double-cut-and-join (DCJ) operation [9], which mimics organizational rearrangements, such as inversions, fusions, fissions and translocations.

In our research group an alternative (NP-hard) *family-free* setting for genome rearrangement approaches was proposed in 2013 [10]. Our studies were further extended, resulting in a model that does not require the pre-computation of gene families and, besides DCJ operations, takes into account insertions and deletions of DNA segments, collectively called *indels* [11, 12]. This model is able to infer pairwise orthologs between two genomes directly, simultaneously based on gene similarities and rearrangements. In practice, its optimization function can be solved exactly due to an ILP formulation [12] that is called $\text{FF-DCJ-I}\textsc{ndel}$ and also reports an optimal matching of orthologs between the two analyzed genomes. (The ILP $\text{FF-DCJ-I}\textsc{ndel}$ is itself based on the previous formulations for family-based approaches [7, 8].)

With these achievements we were able to invert the traditional paradigm of genome rearrangement studies: instead of requiring the gene families to proceed with rearrangement comparisons, it became possible to use rearrangement comparisons for inferring the gene families.^1^ Indeed, in our previous work [13], we did a first attempt of using $\text{FF-DCJ-I}\textsc{ndel}$ for inferring genome-scale gene families across several species. More precisely, given a set of genomes, our method first computes all pairwise optimal gene matchings, which are integrated into gene families in the second step, resulting in a complete pipeline called $\text{O} \textsc{rtho} \text{FFGC}$,^2^ whose inferences displayed good quality in the analysis of completely assembled genomes.

However, the integrated $\text{FF-DCJ-I}\textsc {ndel}$ may not converge in some cases, in particular when the number of segments of a genome is large, e.g. for genomes that are not completely assembled but split in several contigs. The main reason is that each ILP pairwise comparison includes an optimal *capping* that must allow the end of any linear segment of one genome to be matched to the end of any linear segment of the other genome. The optimal capping then produces an exponential increase of the search space.

In this work, we design and implement a heuristic capping algorithm that replaces the optimal capping by clustering (based on their gene content intersections) the linear segments into $m \ge 1$ subsets, so that the ends of the linear segments in the same subset *S* can only be matched to elements of *S*. Furthermore, in each subset, instead of allowing all possible connections, we let only the ends of content-related segments be connected. Although there is no guarantee that *m* is much bigger than one, and with the possible side effect of resulting in sub-optimal instead of optimal gene matchings, the heuristic works very well in practice, from both the speed performance and the quality of computed solutions.

We call $\text{O} \textsc{rtho} \text{FFGC}\!\!\thickapprox$ the new complete pipeline adopting the heuristic capping for $\text{FF-DCJ-I}\textsc {ndel}$. Our experiments on five primate genomes show that for complete assemblies $\text{O} \textsc{rtho} \text{FFGC}\!\!\thickapprox$ reports orthologies that are very similar to those computed by $\text{O} \textsc{rtho} \text{FFGC}$, which is the version of our tool with optimal capping. Moreover, with $\text{O} \textsc{rtho} \text{FFGC}\!\!\thickapprox$ we can efficiently analyze fruit fly genomes with incomplete or heavily fragmented assemblies distributed in hundreds or even thousands of contigs. Considering 11 fruit fly genomes whose assemblies are split into 507 contigs on average and using $\text{F} \textsc {ly} \text{B} \textsc{ase}$ orthologies (https://flybase.org) as reference, we compared the gene families inferred by $\text{O} \textsc{rtho} \text{FFGC}\!\!\thickapprox$ to $\textsc {Oma}$ [14, 15], $\text{P} \textsc {rotein} \text{O} \textsc {rtho}$ [16], and $\textsc {Poff}$ [17]. Our results show that $\text{O} \textsc{rtho} \text{FFGC}\!\!\thickapprox$ inferred a higher number of complete cliques of genes, with a higher intersection with $\text{F} \textsc {ly} \text{B} \textsc{ase}$, when compared to gene families computed by other inference tools.

This paper is an extended version of a work recently presented at WABI [18]. In this extension, besides the already mentioned analysis of five primate genomes, we added a post-processing step that was not included in the work presented at WABI: refining, with the aid of the $\textsc {mcl}$ algorithm [19], our *ambiguous* families (those with more than one gene per genome), which improved even more the accuracy of our results. Another relevant point of the extension presented here is the optimization of several aspects of our implementation, achieving running times that are similar to the fastest alternative tools $\text{P} \textsc {rotein} \text{O} \textsc {rtho}$ and $\textsc {Poff}$ for the *Drosophila* dataset, despite the use of pairwise ILP computations.

## Orthology inference via family-free genome rearrangements

For studying large-scale genome rearrangements a high-level view of a *chromosome* is adopted. In this view each chromosome is represented by a sequence of *genes*. Since each gene is an oriented DNA fragment, we need to distinguish its two possible representations: a gene *g* is represented by the symbol *g* itself, if it is read in direct orientation, or by the symbol $\overline{g}$, if it is read in reverse orientation. In our notation, all genes of a linear chromosome are concatenated in a string that can be read in any of the two directions and is flanked by square brackets. As an example, let $C=[\;\overline{\texttt{6}}\;\texttt{1}\;\texttt{8}\;\texttt{9}\;\overline{\texttt{4}}\;]$ be a linear chromosome. A *genome* is then a set of chromosomes and can be transformed with the following types of mutations:

1. **Structural rearrangements (DCJ operations):** A *cut* performed on a chromosome *C* of a genome ${\mathbb {A}}$ separates two adjacent genes of *C*. A *double-cut and join* or *DCJ* applied on genome ${\mathbb {A}}$ is the operation that performs cuts in two different positions of distinct chromosomes or of the same chromosome of ${\mathbb {A}}$, creating four open ends, and joins these open ends in a different way [1, 9]. For example, let ${\mathbb {A}}=\{\;[\;\overline{\texttt{6}}\;\texttt{1}\;\texttt{8}\;\texttt{9}\;\overline{\texttt{4}}\;], [\;\texttt{3}\;\overline{\texttt{5}}\;\overline{\texttt{7}}\;\texttt{2}\;]\;\}$, and consider a DCJ that cuts between genes $\texttt{1}$ and $\texttt{8}$ of its first chromosome and between genes $\texttt{7}$ and $\texttt{2}$ of its second chromosome, creating segments $\overline{\texttt{6}}\;\texttt{1}\bullet$, $\bullet \texttt{8}\;\texttt{9}\;\overline{\texttt{4}}$, $\texttt{3}\;\overline{\texttt{5}}\;\overline{\texttt{7}}\bullet$ and $\bullet \texttt{2}$ (where the symbols $\bullet$ represent the open ends). If we join the first with the fourth and the second with the third open end, we get ${\mathbb {A}}'=\{\;[\;\overline{\texttt{6}}\;\texttt{1}\;\texttt{2}\;], [\;\texttt{3}\;\overline{\texttt{5}}\;\overline{\texttt{7}}\;\texttt{8}\;\texttt{9}\;\overline{\texttt{4}}\;]\;\}$, that is, the described DCJ operation is a translocation transforming ${\mathbb {A}}$ into ${\mathbb {A}}'$. Indeed, a DCJ operation can correspond not only to a translocation but to several structural rearrangements, such as an inversion, a fusion or a fission.
2. **Content-modifying (indel operations):** The content of a chromosome can be modified with *insertions* and with *deletions* of blocks of contiguous genes, collectively called *indel* operations. Note that at most one chromosome can be entirely deleted or inserted at once. As an example, consider the deletion of segment $\overline{\texttt{7}}\;\texttt{8}\;\texttt{9}$ from chromosome $[\;\texttt{3}\;\overline{\texttt{5}}\;\overline{\texttt{7}}\;\texttt{8}\;\texttt{9}\;\overline{\texttt{4}}\;]$, resulting in chromosome $[\;\texttt{3}\;\overline{\texttt{5}}\;\overline{\texttt{4}}\;]$. A gene cannot be deleted and then reinserted, nor inserted and then deleted. This restriction prevents the *free lunch* artifact of sorting one genome into the other by simply deleting the chromosomes of the first and inserting the chromosomes of the second, ignoring their common parts.

### Computing an optimal set of orthologs between two genomes

We can represent the pairwise similarities between the genes of genome ${\mathbb {A}}$ and the genes of genome ${\mathbb {B}}$ in the so called *gene similarity graph* [10], denoted by $\mathcal {S}({\mathbb {A}}, {\mathbb {B}})$. This is a weighted bipartite graph that has a vertex for each gene in genome ${\mathbb {A}}$ and a vertex for each gene in genome ${\mathbb {B}}$. Furthermore, for each pair of genes $g_1\in {\mathbb {A}},g_2\in {\mathbb {B}}$, denote by $\sigma (g_1, g_2)$ their *normalized similarity*, a value that ranges in the interval [0, 1]. Given a filter $\digamma$, if $\sigma (g_1, g_2)$ is not discarded by $\digamma$, there is an edge *e* connecting $g_1$ and $g_2$ in $\mathcal {S}({\mathbb {A}},{\mathbb {B}})$ whose score is $\sigma (e) = \sigma (g_1, g_2)$. In addition, to each vertex *u* of $\mathcal {S}({\mathbb {A}},{\mathbb {B}})$ we assign a weight *w*(*u*) that can be obtained as follows: $w(u)=\max \{ \sigma (uv) \mid uv \in \mathcal {S}({\mathbb {A}},{\mathbb {B}})\}$, that is, *w*(*u*) is the maximum similarity among the edges incident to the vertex (or gene) *u* in $\mathcal {S}({\mathbb {A}},{\mathbb {B}})$.

A matching ${\mathcal {O}}$ from $\mathcal {S}({\mathbb {A}},{\mathbb {B}})$, here also called an *ortholog-set*, defines the tuple $({\mathbb {A}},{\mathbb {B}},{\mathcal {O}})$, in which every two genes *a*, *b*, such that $a \in {\mathbb {A}}$, $b\in {\mathbb {B}}$ and $ab \in {\mathcal {O}}$, are considered to be *orthologs*. The *complement* of ${\mathcal {O}}$, denoted by $\widetilde{{\mathcal {O}}}$, is the set composed of genes whose corresponding vertices in $\mathcal {S}({\mathbb {A}},{\mathbb {B}})$ are ${\mathcal {O}}$-unsaturated.

**Fig. 1:**
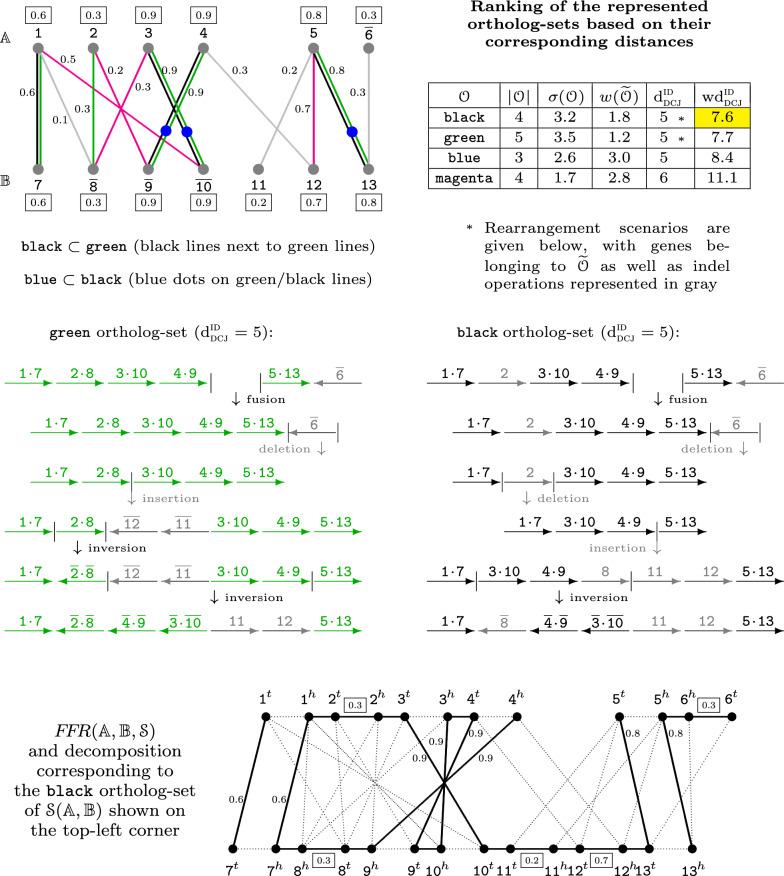
On the top part is displayed the gene similarity graph $\mathcal {S}({\mathbb {A}},{\mathbb {B}})$ of genomes ${\mathbb {A}}=\{\;[\;\texttt{1}\;\;\texttt{2}\;\;\texttt{3}\;\;\texttt{4}\;]~[\;\texttt{5}\;\;\overline{\texttt{6}}\;]\;\}$ and ${\mathbb {B}}=\{\;[\;\texttt{7}\;\;\overline{\texttt{8}}\;\;\overline{\texttt{9}}\;\;\overline{\texttt{10}}\;\;\texttt{11}\;\;\texttt{12}\;\;\texttt{13}\;]\;\}$ and next to it a table with the ranking of four distinct ortholog-sets. On the middle the rearrangement scenarios induced by two of these ortholog-sets are shown. On the bottom part the family-free relational graph $F\!F\!R({\mathbb {A}},{\mathbb {B}},\mathcal {S})$ is illustrated, highlighting the edges of the decomposition corresponding to the (black) ortholog-set ${\mathcal {O}}=\{\{\texttt{1},\texttt{7}\}$, $\{\texttt{3},\texttt{10}\}, \{\texttt{4},\texttt{9}\}, \{\texttt{5},\texttt{13}\}\}$. (This decomposition has two ${\mathbb {A}}{\mathbb {B}}$-paths, one ${\mathbb {A}}{\mathbb {A}}$-path and one cycle.) All extremity and indel edges in $F\!F\!R({\mathbb {A}},{\mathbb {B}},\mathcal {S})$ are respectively scored and weighted according to $\mathcal {S}({\mathbb {A}},{\mathbb {B}})$ but the scores and weights of edges not derived from ${\mathcal {O}}$ or $\widetilde{{\mathcal {O}}}$ are omitted

The DCJ-indel distance $\textrm{d} _\textsc {dcj} ^\textsc {id} ({\mathbb {A}},{\mathbb {B}},{\mathcal {O}})$ is the minimum number of DCJ and indel operations required to transform ${\mathbb {A}}$ into ${\mathbb {B}}$ assuming the orthologs given by ${\mathcal {O}}$ and allowing only the genes belonging to the complement $\widetilde{{\mathcal {O}}}$ to be inserted or deleted. It can be computed using an approach relying on the cycles and paths of a graph that represents the structural relation between genomes ${\mathbb {A}}$ and ${\mathbb {B}}$ according to the ortholog-set ${\mathcal {O}}$ [3, 12] (this graph is equivalent to a consistent decomposition of the family-free relational graph, described in the next subsection and represented in Fig. 1 (bottom)). Together with the scores of edges and vertices of $\mathcal {S}({\mathbb {A}},{\mathbb {B}})$, the DCJ-indel distance $\textrm{d} _\textsc {dcj} ^\textsc {id}$ allows the computation of the weighted rearrangement distance $\textrm{wd} _\textsc {dcj}^\textsc {id}$ [12]:

$$\begin{aligned} \textrm{wd} _\textsc {dcj}^\textsc {id}({\mathbb {A}},{\mathbb {B}},\mathcal {S},{\mathcal {O}})=\textrm{d} _\textsc {dcj} ^\textsc {id} ({\mathbb {A}},{\mathbb {B}},{\mathcal {O}})+|{\mathcal {O}}|-\sigma ({\mathcal {O}})+w(\widetilde{{\mathcal {O}}}), \end{aligned}$$

where

$$\begin{aligned} \sigma ({\mathcal {O}})=\sum _{e\in {\mathcal {O}}} \sigma (e) \text { and } w(\widetilde{{\mathcal {O}}})=\sum _{v\in \widetilde{{\mathcal {O}}}} w(v). \end{aligned}$$

Then, given that ${\mathfrak {M}}$ is the set of all possible ortholog-sets in $\mathcal {S}({\mathbb {A}},{\mathbb {B}})$, the rearrangement distance between ${\mathbb {A}}$ and ${\mathbb {B}}$ is the result of the following optimization:

$$\begin{aligned} \textsc {GenDiFF}({\mathbb {A}}, {\mathbb {B}}, \mathcal {S})=\min _{ {\mathcal {O}}\in {\mathfrak {M}} }\{ \textrm{wd} _\textsc {dcj}^\textsc {id}({\mathbb {A}},{\mathbb {B}},\mathcal {S},{\mathcal {O}}) \} \,. \end{aligned}$$

Figure 1 shows examples of ortholog-sets and their distances. Denote by $\textsc {OrthoFF}({\mathbb {A}}, {\mathbb {B}}, \mathcal {S})$ an *optimal* ortholog-set in $\mathcal {S}({\mathbb {A}},{\mathbb {B}})$, which is an ortholog-set whose rearrangement distance equals $\textsc {GenDiFF}({\mathbb {A}}, {\mathbb {B}}, \mathcal {S})$. Computing the rearrangement distance $\textsc {GenDiFF}({\mathbb {A}}, {\mathbb {B}}, \mathcal {S})$ and finding an optimal ortholog-set $\textsc {OrthoFF}({\mathbb {A}}, {\mathbb {B}}, \mathcal {S})$ are NP-hard problems [12].

### Family-free relational graph

For solving both NP-hard problems $\textsc {GenDiFF}({\mathbb {A}}, {\mathbb {B}}, \mathcal {S})$ and $\textsc {OrthoFF}({\mathbb {A}}, {\mathbb {B}}, \mathcal {S})$ we adopt the approach of decomposing the following graph.

The *family-free relational graph* $F\!F\!R({\mathbb {A}},{\mathbb {B}},\mathcal {S})$, shown in Fig. 1 (bottom), represents all possible weighted distances corresponding to all candidate ortholog-sets in $\mathcal {S}({\mathbb {A}},{\mathbb {B}})$ [12]. Given a gene *m*, denote the extremities of *m* by $m^{\;\!h}$ (*head*) and $m^{\;\!t}$ (*tail*). The graph $F\!F\!R({\mathbb {A}},{\mathbb {B}},\mathcal {S})$ has a set $V({\mathbb {A}})$ with a vertex for each of the two extremities of each gene of genome ${\mathbb {A}}$ and a set $V({\mathbb {B}})$ with a vertex for each of the two extremities of each gene of genome ${\mathbb {B}}$.

The set of edges is partitioned into several subsets:

- Sets $E_{\text {adj}}^{{\mathbb {A}}}$ and $E_{\text {adj}}^{{\mathbb {B}}}$ contain adjacency edges connecting adjacent extremities of genes in ${\mathbb {A}}$ and in ${\mathbb {B}}$.
- The set $E_{\gamma }$ contains, for each edge $ab \in \mathcal {S}({\mathbb {A}},{\mathbb {B}})$, an extremity edge connecting $a^{\;\!t}$ to $b^{\;\!t}$, and an extremity edge connecting $a^{\;\!h}$ to $b^{\;\!h}$. To both edges $a^{\;\!t}b^{\;\!t}$ and $a^{\;\!h}b^{\;\!h}$, that are called *siblings*, we assign the same score $\sigma (ab)$ of the edge *ab* in $\mathcal {S}({\mathbb {A}},{\mathbb {B}})$: $\sigma (a^{\;\!t}b^{\;\!t})=\sigma (a^{\;\!h}b^{\;\!h})=\sigma (ab)$.
- Sets $E_{\text {id}}^{{\mathbb {A}}}$ and $E_{\text {id}}^{{\mathbb {B}}}$ contain indel edges connecting the two extremities of each gene in ${\mathbb {A}}$ and in ${\mathbb {B}}$. Each indel edge $m^{\;\!h}m^{\;\!t}$ receives the weight *w*(*m*) of vertex *m* in $\mathcal {S}({\mathbb {A}},{\mathbb {B}})$: $w(m^{\;\!h}m^{\;\!t})=w(m)$. (Recall that *w*(*m*) is the maximum score among the edges incident to *m* in $\mathcal {S}({\mathbb {A}},{\mathbb {B}})$.)

### Consistent decompositions of the family-free relational graph

A *decomposition* of $F\!F\!R({\mathbb {A}},{\mathbb {B}},\mathcal {S})$ is a collection of edges building vertex-disjoint components, that can be cycles and/or paths, covering all vertices of $F\!F\!R({\mathbb {A}},{\mathbb {B}},\mathcal {S})$. A decomposition must be *consistent*, implying that it is *induced* by a set of edges ${\mathcal {L}}$ exclusively composed of pairs of siblings and without any pair of incident edges. The set ${\mathcal {L}}$ is called a *sibling-set* and corresponds to one precise ortholog-set of $\mathcal {S}({\mathbb {A}},{\mathbb {B}})$, denoted by ${\mathcal {O}}_{\mathcal {L}}$. Note that $|{\mathcal {L}}| = 2|{\mathcal {O}}_{\mathcal {L}}|$ and $\sigma ({\mathcal {L}})=2\sigma ({\mathcal {O}}_{\mathcal {L}})$. The *complement* of ${\mathcal {L}}$, denoted by $\widetilde{{\mathcal {L}}}$, is composed of the indel-edges corresponding to the genes of $\widetilde{{\mathcal {O}}}_{\mathcal {L}}$ (the complement of ${\mathcal {O}}_{\mathcal {L}}$), therefore $|\widetilde{{\mathcal {L}}}|=|\widetilde{{\mathcal {O}}}_{\mathcal {L}}|$ and $w(\widetilde{{\mathcal {L}}})=w(\widetilde{{\mathcal {O}}}_{\mathcal {L}})$. The consistent decomposition induced by ${\mathcal {L}}$ is denoted by $D[{\mathcal {L}}]$ and corresponds to:

$$\begin{aligned} D[{\mathcal {L}}]= {\mathcal {L}}\cup \widetilde{{\mathcal {L}}}\cup E_{\text {adj}}^{{\mathbb {A}}} \cup E_{\text {adj}}^{{\mathbb {B}}}. \end{aligned}$$

The consistent decomposition $D[{\mathcal {L}}]$ covers all vertices of $F\!F\!R({\mathbb {A}},{\mathbb {B}},\mathcal {S})$ and is composed of cycles and paths. The paths connect the ends of linear chromosomes in both genomes and can be of three types: either ${\mathbb {A}}{\mathbb {A}}$-path, or ${\mathbb {B}}{\mathbb {B}}$-path or ${\mathbb {A}}{\mathbb {B}}$-path.

The structure of $D[{\mathcal {L}}]$ has all necessary information for computing the value $\textrm{wd} _\textsc {dcj}^\textsc {id}({\mathbb {A}},{\mathbb {B}},\mathcal {S},{\mathcal {O}}_{\mathcal {L}})$, therefore we can say that $\textrm{wd} _\textsc {dcj}^\textsc {id}({\mathbb {A}},{\mathbb {B}},\mathcal {S},{\mathcal {O}}_{\mathcal {L}})=\textrm{wd} _\textsc {dcj}^\textsc {id}(D[{\mathcal {L}}])$ [12]. Given that ${\mathfrak {S}}$ is the set of all possible sibling-sets in $F\!F\!R({\mathbb {A}},{\mathbb {B}},\mathcal {S})$, we can modify our optimization problem to

$$\begin{aligned} \textsc {GenDiFF}({\mathbb {A}}, {\mathbb {B}}, \mathcal {S}) = \displaystyle \min _{{\mathcal {L}}\in {\mathfrak {S}}}\{\textrm{wd} _\textsc {dcj}^\textsc {id}(D[{\mathcal {L}}])\}. \end{aligned}$$

Assuming that a sibling-set ${\mathcal {L}}_\star$ gives the optimal solution for the problem $\textsc {GenDiFF}({\mathbb {A}}, {\mathbb {B}}, \mathcal {S})$, then $\textsc {OrthoFF}({\mathbb {A}}, {\mathbb {B}}, \mathcal {S}) = {\mathcal {O}}_{{\mathcal {L}}_\star }$.

## Capping

The end of a linear chromosome is called *telomere*. The telomeres are also the ends of the paths of any consistent decomposition. Therefore, if $\kappa ({\mathbb {A}})$ is the number of linear chromosomes in ${\mathbb {A}}$ and $\kappa ({\mathbb {B}})$ is the number of linear chromosomes in ${\mathbb {B}}$ the number of paths in any decomposition is $\kappa ({\mathbb {A}})+\kappa ({\mathbb {B}})$. Our ILP is able to capture all necessary properties from the cycles of a decomposition, but cannot handle paths. A way to overcome this problem is by linking all paths of any decomposition with a known technique called *capping* [2].

### Capping a consistent decomposition

The idea of the capping is to split the telomeres into disjoint pairs and then to connect the two elements of each pair, so that all paths are linked into cycles. The only restriction is that a pair cannot contain telomeres from the same genome, therefore, if the numbers of telomeres in the two genomes are different, some *dummy* telomeres need to be created, as we describe in the following.

Suppose that $D[{\mathcal {L}}]$ is any consistent decomposition of $F\!F\!R({\mathbb {A}},{\mathbb {B}},\mathcal {S})$. For each telomere (vertex) *v*, add to $D[{\mathcal {L}}]$ a *cap vertex* $\theta _v$ and connect *v* to $\theta _v$ by an adjacency edge. Now let $\theta ({\mathbb {A}})$ (respectively $\theta ({\mathbb {B}})$) be the set of all cap vertices in ${\mathbb {A}}$ (respectively in ${\mathbb {B}}$). Note that, since each linear chromosome has two ends, the cardinalities of these sets must be even. Moreover, if $|\theta ({\mathbb {A}})|\ne |\theta ({\mathbb {B}})|$, the cardinalities of these sets must be equalized. Let $p_*=\max \{\kappa ({\mathbb {A}}),\kappa ({\mathbb {B}})\}$ and $a_*=|\kappa ({\mathbb {A}})-\kappa ({\mathbb {B}})|$. For equalizing the cardinalities with the minimum number of extra vertices, we need to add $2a_*$ extra cap vertices to the set with smaller cardinality. These extra cap vertices must be split into pairs (arbitrarily chosen) so that the vertices of each pair are connected by a *dummy* adjacency edge in $D[{\mathcal {L}}]$. Denote by ${\hat{\theta }}({\mathbb {A}})$ and ${\hat{\theta }}({\mathbb {B}})$ the sets with equalized cardinalities and let *P* be a *capping-set*, which is a perfect matching between them: for $\gamma \in {\hat{\theta }}({\mathbb {A}})$ and $\gamma ' \in {\hat{\theta }}({\mathbb {B}})$, if $\gamma \gamma ' \in P$, then $\gamma$ and $\gamma '$ are connected by a *cap edge*. Let ${\widehat{D}}[{\mathcal {L}},P]$ be a *capped decomposition* of $D[{\mathcal {L}}]$ with capping-set *P*. It is easy to see that ${\widehat{D}}[{\mathcal {L}},P]$ is composed of cycles only.

#### Optimal capping

**Fig. 2:**
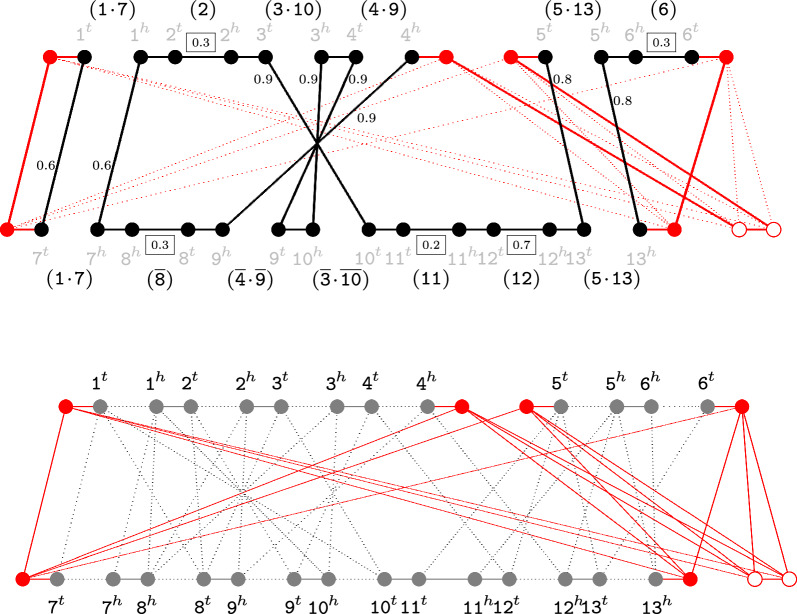
On the top part we show the capping of the decomposition corresponding to the (black) ortholog-set ${\mathcal {O}}=\{\{\texttt{1},\texttt{7}\}$, $\{\texttt{3},\texttt{10}\}, \{\texttt{4},\texttt{9}\}, \{\texttt{5},\texttt{13}\}\}$ from the gene similarity graph $\mathcal {S}({\mathbb {A}},{\mathbb {B}})$ of Fig. 1 (bottom). Each red vertex is a cap vertex. Each filled (red) vertex is connected to a telomere (chromosome/path ends). The unfilled vertices represent the extra (equalizing) vertices connected by a dummy adjacency. The capping is a perfect matching of the complete bipartite graph of the cap vertices. The optimal capping for this decomposition is highlighted. It closes each of its paths into a separate cycle. (In general, an optimal capping of a decomposition may link up to 4 paths into a single cycle [8]). On the bottom part is displayed the complete family-free graph $F\!F\!R({\mathbb {A}},{\mathbb {B}},\mathcal {S})$ optimally capped. Cap edges are unweighted. Scores of extremity edges and weights of indel edges are omitted

So far we explained how to guarantee that all paths in any decomposition are linked into cycles. Note, however, that there are $(2p_*)!$ ways of completely matching the vertices of sets ${\hat{\theta }}({\mathbb {A}})$ and ${\hat{\theta }}({\mathbb {B}})$. For a given decomposition $D[{\mathcal {L}}]$, any of these possibilities, say capping-set *P*, would produce a capped decomposition ${\widehat{D}}[{\mathcal {L}},P]$, and capping the same $D[{\mathcal {L}}]$ with distinct capping-sets may produce distinct weighted costs. Let $P_{\mathcal {L}}$ be an optimal capping-set for $D[{\mathcal {L}}]$, that is, ${\widehat{D}}[{\mathcal {L}},P_{\mathcal {L}}]$ has the minimum weighted cost among all cappings of $D[{\mathcal {L}}]$.^3^ In a previous work we have shown that $\textrm{wd} _\textsc {dcj}^\textsc {id}({\widehat{D}}[{\mathcal {L}},P_{\mathcal {L}}]) = \textrm{wd} _\textsc {dcj}^\textsc {id}(D[{\mathcal {L}}])$ [12]. Therefore, for a consistent decomposition $D[{\mathcal {L}}]$, any of its optimal capping-sets preserves the weighted cost of $D[{\mathcal {L}}]$, reporting both $\textrm{d} _\textsc {dcj} ^\textsc {id} ({\mathbb {A}},{\mathbb {B}},{\mathcal {O}}_{\mathcal {L}})$ and $\textrm{wd} _\textsc {dcj}^\textsc {id}({\mathbb {A}},{\mathbb {B}},\mathcal {S},{\mathcal {O}}_{\mathcal {L}})$. Figure 2 (top) highlights an optimal capping of a consistent decomposition.

### Optimally capped family-free relational graph

All consistent decompositions share the same telomeres, therefore a set of capping-sets for one decomposition is also a set of capping-sets of any other decomposition. If we then simply add all possible capping-sets to the family-free relational graph, which implies adding a complete bipartite graph with partite sets ${\hat{\theta }}({\mathbb {A}})$ and ${\hat{\theta }}({\mathbb {B}})$, we guarantee that an optimal solution can be found. Let the so-called optimal capping (represented in Fig. 2 (bottom)) of $F\!F\!R({\mathbb {A}},{\mathbb {B}},\mathcal {S})$ with the minimum number of extra elements be denoted by $\theta _\star (F\!F\!R({\mathbb {A}},{\mathbb {B}},\mathcal {S}))$ and be defined as follows:

($$1_\star$$)
Add the set of *cap vertices* ${\hat{\theta }}({\mathbb {A}}) = \theta _{{\mathbb {A}}}^{1},\theta _{{\mathbb {A}}}^{2},\ldots ,\theta _{{\mathbb {A}}}^{2p_*}$ and connect each telomere of genome ${\mathbb {A}}$ to one of these cap vertices by an adjacency edge added to $E_{\text {adj}}^{{\mathbb {A}}}$.
($$2_\star$$)
Similarly, add the set of cap vertices ${\hat{\theta }}({\mathbb {B}}) = \theta _{{\mathbb {B}}}^{1}, \theta _{{\mathbb {B}}}^{2},\ldots ,\theta _{{\mathbb {B}}}^{2p_*}$ and connect each telomere of genome ${\mathbb {B}}$ to one of these cap vertices by an adjacency edge added to $E_{\text {adj}}^{{\mathbb {B}}}$.
($$3_\star$$)
Add (arbitrarily chosen) $p_* - \kappa ({\mathbb {A}})$ dummy adjacency edges to $E_{\text {adj}}^{{\mathbb {A}}}$ and $p_* - \kappa ({\mathbb {B}})$ dummy adjacency edges to $E_{\text {adj}}^{{\mathbb {B}}}$. (Note that only one of the two genomes may have dummy adjacencies.)
($$4_\star$$)
Connect all cap vertices in ${\hat{\theta }}({\mathbb {A}})$ to all cap vertices in ${\hat{\theta }}({\mathbb {B}})$ with *cap edges*. The set of all cap edges is denoted by $E_{\theta }$.

Since all $2p_*$ cap vertices in ${\mathbb {A}}$ are connected to all $2p_*$ cap vertices in ${\mathbb {B}}$ and any perfect matching of these edges is a valid capping, the search space of our optimization problem is multiplied by $(2p_*)!$. Denote by ${\mathfrak {P}}$ the set of all possible capping-sets (perfect matchings) between the vertices from ${\hat{\theta }}({\mathbb {A}})$ and ${\hat{\theta }}({\mathbb {B}})$. The optimization problem over $\theta _\star (F\!F\!R({\mathbb {A}},{\mathbb {B}},\mathcal {S}))$ can be rewritten as

$$\begin{aligned} \textsc {GenDiFF}({\mathbb {A}}, {\mathbb {B}}, \mathcal {S}) = \displaystyle \min _{{\mathcal {L}}\in {\mathfrak {S}}, P\in {\mathfrak {P}}}\{\textrm{wd} _\textsc {dcj}^\textsc {id}({\widehat{D}}[{\mathcal {L}},P]\}. \end{aligned}$$

Assuming that a sibling-set ${\mathcal {L}}_\star$ (together with one of its optimal capping-sets) gives the optimal solution for $\textsc {GenDiFF}({\mathbb {A}}, {\mathbb {B}}, \mathcal {S})$, an optimal ortholog-set is $\textsc {OrthoFF}({\mathbb {A}}, {\mathbb {B}}, \mathcal {S}) = {\mathcal {O}}_{{\mathcal {L}}_\star }$. Both problems $\textsc {GenDiFF}$ and $\text{O} \textsc{rtho} \text{FF}$ can be solved with the ILP formulation $\text{FF-DCJ-I}\textsc {ndel}$ [12], which can be found in Additional file 1: Section (A).

### Integration of pairwise optimal ortholog-sets into gene families

In our previous work [13], the ILP $\text{FF-DCJ-I}\textsc {ndel}$ solving $\text{O} \textsc{rtho} \text{FF}$ (with optimal capping) was integrated in a tool called $\text{O} \textsc{rtho} \text{FFGC}$ for inferring gene families across several species. The pipeline can be summarized as follows: given a set of *n* genomes, gene similarities and ortholog-sets are computed for all pairwise comparisons and simply integrated into an *n*-partite graph. The connected components of this graph are the inferred gene families.

In this work we modify this pipeline by replacing the optimal capping by a heuristic lighter one, as we explain in the next section.

## Heuristic capping

Conceptually our approach can handle genomes with several linear chromosomes and even partially assembled genomes distributed into many contigs/scaffolds: each of these is a linear segment and could simply be treated as the same object that we so far called chromosome. However, as already explained, the optimal capping multiplies the search space of $F\!F\!R({\mathbb {A}},{\mathbb {B}})$ by $(2p_*)!$ where $p_*$ is the maximum between the number of linear segments in genomes ${\mathbb {A}}$ and ${\mathbb {B}}$. This effect makes it unfeasible to analyze genomes with a large number of segments with our ILP over an optimally capped family-free relational graph.

**Fig. 3:**
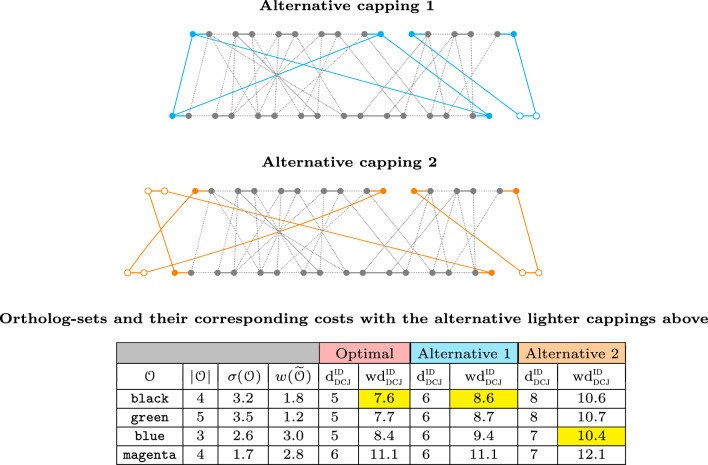
Examples of two arbitrary lighter valid cappings of the family-free relational diagram from Fig. 1 (bottom) and their effects on the ranking of ortholog-sets/sibling-sets represented in Fig. 1 (top). Both cappings affect the computed distances, but, while the capping shown in the left (cyan) preserves the optimal ranking, the one shown in the right (orange) does not

One way of overcoming this issue is by adopting a lighter capping, for example by removing some edges from the complete bipartite graph of ${\hat{\theta }}({\mathbb {A}})$ and ${\hat{\theta }}({\mathbb {B}})$, and/or by partitioning these sets into subsets that are capped independently. In any case it is important to guarantee that a capping is *valid*, that is, it allows to find a capping-set (a perfect matching of the cap vertices). A valid lighter capping may not include the optimal capping-sets, and therefore may not preserve the computed weighted costs. Note, however, that even if the weighted costs are not preserved, the ranking of the ortholog-sets/sibling-sets may not be affected. In Fig. 3 we show examples of arbitrary lighter valid cappings and their effects on the ortholog-set ranking.

### Perfect shared-content graph with thresholds $\tau$ and $\epsilon$

Our goal is therefore to develop a lighter heuristic capping that may potentially preserve the original (optimal) ranking of the best ortholog-sets/sibling-sets. We achieve this by connecting cap vertices only between the telomeres of the linear segments (contigs or chromosomes) that (potentially) share most of their genomic contents. This is because those telomeres have a higher chance of being in the same paths of the best consistent decompositions of the family-free relational graph.

Given two linear segments $A \in {\mathbb {A}}$ and $B \in {\mathbb {B}}$, let their *shared genomic content* $\Omega (A,B)$ be the sum of the scores of edges in $\mathcal {S}$ connecting genes from *A* to genes from *B*. Formally, for $A \subseteq {\mathbb {A}}$, $B \subseteq {\mathbb {B}}$, we have

$$\begin{aligned} \Omega (A,B) = \displaystyle \sum _{\begin{array}{c} g_{1}g_{2} \in \mathcal {S}\\ g_1 \in A\\ g_2 \in B \end{array}} \sigma (g_1g_2). \end{aligned}$$

Now let $\mathcal {C}({\mathbb {A}},{\mathbb {B}}) = (U_{\mathbb {A}}, U_{\mathbb {B}}, F)$ be the bipartite *shared-content graph* where the vertex sets are

$$\begin{aligned} \begin{array}{ccll} U_{\mathbb {A}} &{} = &{} \{A: A \text { is a linear segment in } {\mathbb {A}}\} &{}\text { and }\\ U_{\mathbb {B}} &{} = &{} \{B: B \text { is a linear segment in } {\mathbb {B}}\}&{}. \end{array} \end{aligned}$$

Initially the set of edges is $F = \{ AB \mid A \in U_{\mathbb {A}}, B \in U_{\mathbb {B}} \text { and } \Omega (A,B) > 0\}$. Each edge *AB* has a score $\Omega (A,B)$. We then reduce the size of $\mathcal {C}({\mathbb {A}},{\mathbb {B}})= (U_{\mathbb {A}}, U_{\mathbb {B}}, F)$, by removing edges based on two parameters:

(i) Given a positive integer $\tau$, we remove edges from *F* by applying a filtering procedure that simply iterates over $U_{\mathbb {A}}\cup U_{\mathbb {B}}$, keeping in *F* only the $\tau$ edges of highest scores for each vertex.
(ii) Then, given a rational threshold $\epsilon \in (0,1]$, the remaining edges are again filtered out to remove weak relations between linear segments: let the score of a vertex $v \in \mathcal {C}({\mathbb {A}},{\mathbb {B}})$ be $\Omega (v)=\displaystyle \max _{uv \in F}\{\Omega (u,v)\}$; the edges inciding on *v* that have scores below $\epsilon \;\Omega (v)$ are removed.

#### Capping attempt induced by the shared-content graph

The shared-content graph will now *induce* our capping procedure. The idea is to allow cap connections only between the ends of linear segments that are connected in $\mathcal {C}$. Therefore, the linear segments that are in the same connected component of $\mathcal {C}$ will be capped together, independently from the linear segments that are in other connected components. In other words, the connected components of $\mathcal {C}$ will impose a partitioning of the capping procedure.

**Fig. 4:**
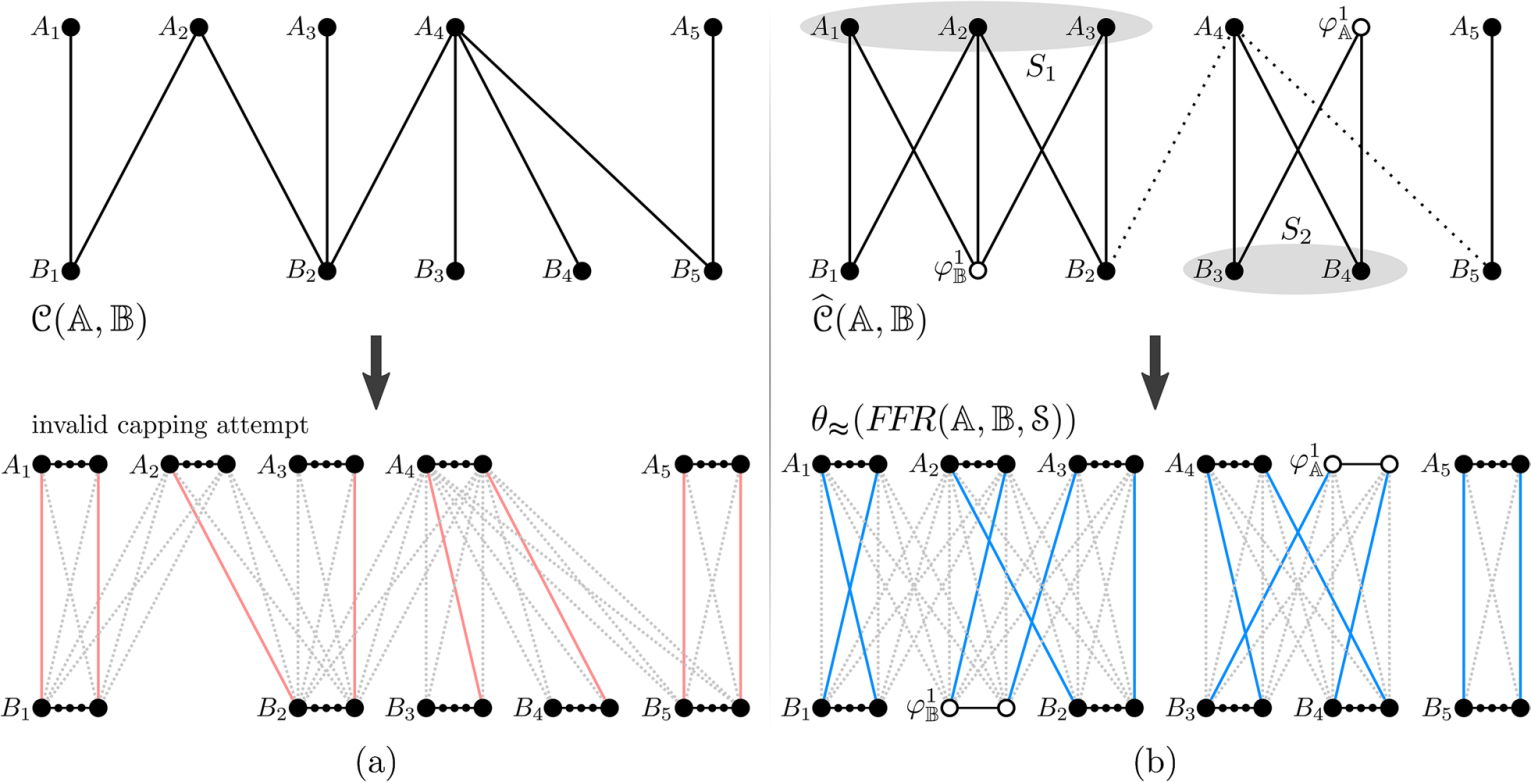
**a** Example of a shared-content graph $\mathcal {C}({\mathbb {A}},{\mathbb {B}})$ with omitted edge scores. The genomes ${\mathbb {A}}$ and ${\mathbb {B}}$ have linear segments $A_{1{..}{}5}$ and $B_{1{..}{}5}$, respectively. The capping of $F\!F\!R({\mathbb {A}},{\mathbb {B}},\mathcal {S})$ induced by $\mathcal {C}$ is invalid. **b** Transformation of $\mathcal {C}$ into a perfect shared-content graph ${\widehat{\mathcal {C}}}({\mathbb {A}},{\mathbb {B}})$: vertex sets $S_1$ and $S_2$ represent Hall violators (among other possibilities) that demand the creation of dummy segments $\varphi _{\mathbb {B}}^1$ and $\varphi _{\mathbb {A}}^1$, respectively. Dotted edges represent those that are non-matchable and must be removed from ${\widehat{\mathcal {C}}}$ after the completion is finished. Notice that the component with vertices $A_1, A_2, A_3, B_1, \varphi _{\mathbb {B}}^1 \text { and } B_2$ is not a complete bipartite subgraph. (In both (**a**, **b**), we give an abstract illustration of the capped $F\!F\!R$ where only cap vertices, cap edges and dummy adjacencies are represented explicitly, while vertices of gene extremities between cap vertices are represented by a line with small dots. In addition, colored solid edges represent a maximum cardinality matching between cap vertices, while the cap edges not in the matching are dashed grey)

Let us then assume that $\mathcal {C}$ has a single connected component. Note that a capping induced by $\mathcal {C}$ can only be valid if its partite sets $U_{\mathbb {A}}$ and $U_{\mathbb {B}}$ are of the same size. This necessary condition also applies and is sufficient for the optimal capping, but here it is not sufficient: even when $U_{\mathbb {A}}$ and $U_{\mathbb {B}}$ are of the same size, since not all connections between the ends of the linear segments in $U_{\mathbb {A}}$ and in $U_{\mathbb {B}}$ are present, the induced capping could be invalid (Fig. 4a). Here the necessary and sufficient condition for a valid capping is the existence of a perfect matching in $\mathcal {C}({\mathbb {A}},{\mathbb {B}})$, as stated in Lemma 1, whose proof relies on a theorem closely related to perfect matchings and demonstrated by Hall [20] in 1935. Denote by ${\mathcal {N}}(S)$ the neighborhood of a vertex set *S*, that is, the set of all vertices adjacent to some vertex of *S*.

##### Theorem 1

**(Hall’s marriage theorem)** Let $G=(U,V,E)$ be a bipartite graph. There exists a matching in *G* that covers the vertex set *V* if and only if for each subset $S \subseteq V$, $|S| \le |{\mathcal {N}}(S)|$.

Note that a perfect matching exists in $\mathcal {C}$ if and only if the condition of Theorem 1 holds for both $V_{\mathbb {A}}$ and $V_{\mathbb {B}}$. We can now establish the relation between perfect matchings in $\mathcal {C}$ and the validity of the capping it induces in the family-free relational graph.

##### Lemma 1

A perfect matching exists in $\mathcal {C}({\mathbb {A}},{\mathbb {B}})$ if and only if the capping of $F\!F\!R({\mathbb {A}},{\mathbb {B}},\mathcal {S})$ induced by $\mathcal {C}$ is valid.

##### Proof

If a perfect matching *M* exists in $\mathcal {C}$, for each edge *AB* in *M*, in the induced capping of $F\!F\!R({\mathbb {A}},{\mathbb {B}},\mathcal {S})$ the pair of cap vertices connected to the ends of *A* can be connected in any of the two distinct ways to the pair of cap vertices connected to the ends of *B*, resulting in a capping-set.

The converse is shown by contraposition. Suppose that a maximum cardinality matching *M* in $\mathcal {C}$ is not a perfect matching. Therefore, by Hall’s marriage theorem, there exists some *S* in $\mathcal {C}$ such that $|{\mathcal {N}}(S)| < |S|$. Let $S'$ be the set of cap vertices in the capping induced by $\mathcal {C}$ for all linear segments in *S*. Since the connection of these cap vertices follows $\mathcal {C}$ and each linear segment has two cap vertices, it is clear that $|S'| = 2|S|$ and $|{\mathcal {N}}(S')| = 2|{\mathcal {N}}(S)|$, hence, $|{\mathcal {N}}(S')| < |S'|$. By the pigeonhole principle, at least 2 cap vertices (because $|{\mathcal {N}}(S')|$ and $|S'|$ are even numbers) will not be incident to any cap edge, therefore no capping-set exists. $\square$

#### Building the perfect shared-content graph

We will now describe a procedure for transforming the shared-content graph $\mathcal {C}({\mathbb {A}},{\mathbb {B}})$ into a *perfect shared-content graph* ${\widehat{\mathcal {C}}}({\mathbb {A}},{\mathbb {B}})$ that has at least one perfect matching, as shown in Algorithm 1. In the completion loop, dummy segments are iteratively created until a perfect matching is possible. If a maximum cardinality matching *M* is found but is not a perfect matching, a Hall violator set *S* can be found as follows. Let *v* be a vertex unsaturated by *M*, then $S = \{v\} \cup \{u \mid u$ is reachable from *v* by an *M*-alternating path$\}$. Finally, $|S| - |{\mathcal {N}}(S)|$ dummy segments are created and connected to each linear segment in *S*.
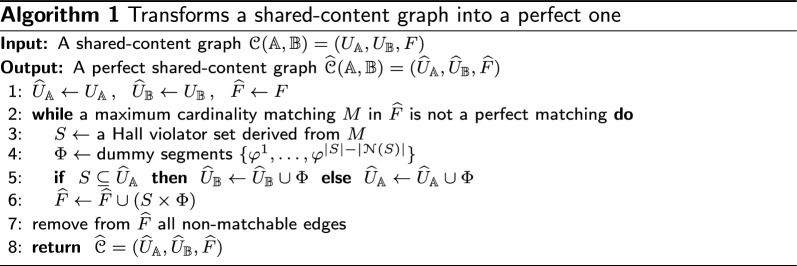


An edge in ${\widehat{\mathcal {C}}}({\mathbb {A}},{\mathbb {B}})$ is *matchable* if it is part of at least one perfect matching and *non-matchable* otherwise. Once the completion loop is finished, ${\widehat{\mathcal {C}}}$ admits at least one perfect matching and its matchable edges can be identified efficiently [21]. The last step of Algorithm 1 is then removing from ${\widehat{\mathcal {C}}}$ all non-matchable edges. An example of the construction of a perfect shared-content graph is illustrated above, in Fig. 4b.

### Heuristically capped family-free relational graph

The bipartite perfect shared-content graph ${\widehat{\mathcal {C}}}({\mathbb {A}},{\mathbb {B}})$ has edge set ${\widehat{F}}$ and partite sets ${\widehat{U}}_{\mathbb {A}} = U_{\mathbb {A}} \cup U^\varphi _{\mathbb {A}}$ and ${\widehat{U}}_{\mathbb {B}} = U_{\mathbb {B}} \cup U^\varphi _{\mathbb {B}}$, where $U_{\mathbb {A}}$ and $U_{\mathbb {B}}$ are the sets of linear segments and $U^\varphi _{\mathbb {A}}$ and $U^\varphi _{\mathbb {B}}$ the sets of dummy segments. Recall that the sets ${\widehat{U}}_{\mathbb {A}}$ and ${\widehat{U}}_{\mathbb {B}}$ have the same cardinality, which here we denote by $p_\thickapprox$. The heuristic capping $\theta _\thickapprox$ of the family-free relational graph $F\!F\!R({\mathbb {A}},{\mathbb {B}},\mathcal {S})$ induced by ${\widehat{\mathcal {C}}}({\mathbb {A}},{\mathbb {B}})$ is shown in Fig. 4b and described as follows:

($$1_\thickapprox$$)
Add the set of cap vertices ${\hat{\theta }}({\mathbb {A}}) = \theta _{{\mathbb {A}}}^{1},\theta _{{\mathbb {A}}}^{2},\ldots ,\theta _{{\mathbb {A}}}^{2p_\thickapprox }$. For $i = 1 \ldots |U_{\mathbb {A}}|$, associate each linear segment $A_i \in U_{\mathbb {A}}$ to cap vertices $\theta _{{\mathbb {A}}}^{2i-1}$ and $\theta _{{\mathbb {A}}}^{2i}$ and connect with adjacency edges one telomere of $A_i$ to $\theta _{{\mathbb {A}}}^{2i-1}$ and the other to $\theta _{{\mathbb {A}}}^{2i}$. Note that $2|U_{\mathbb {A}}^\varphi |$ cap vertices remain disconnected.
($$2_\thickapprox$$)
Similarly, add cap vertices ${\hat{\theta }}({\mathbb {B}}) = \theta _{{\mathbb {B}}}^{1}, \theta _{{\mathbb {B}}}^{2},\ldots ,\theta _{{\mathbb {B}}}^{2p_\thickapprox }$. For $j = 1 \ldots |V_{\mathbb {B}}|$, associate each linear segment $B_j \in U_{\mathbb {B}}$ to cap vertices $\theta _{{\mathbb {B}}}^{2j-1}$ and $\theta _{{\mathbb {B}}}^{2j}$ and connect with adjacency edges one telomere of $B_j$ to $\theta _{{\mathbb {B}}}^{2j-1}$ and the other to $\theta _{{\mathbb {B}}}^{2j}$. Again, $2|U_{\mathbb {B}}^\varphi |$ cap vertices remain disconnected.
($$3_\thickapprox$$)
For $i_\circ = 1 \ldots |U_{\mathbb {A}}^\varphi |$ and $i = |U_{\mathbb {A}}|+i_\circ$, connect the pair of cap vertices $\theta _{{\mathbb {A}}}^{2i-1}$ and $\theta _{{\mathbb {A}}}^{2i}$ by a dummy adjacency edge, associating this pair to the dummy segment $\varphi ^{i_\circ }_{\mathbb {A}} \in U_{\mathbb {A}}^\varphi$. Similarly, for $j_\circ = 1 \ldots |U_{\mathbb {B}}^\varphi |$ and $j = |U_{\mathbb {B}}|+j_\circ$, connect the pair of cap vertices $\theta _{{\mathbb {B}}}^{2j-1}$ and $\theta _{{\mathbb {B}}}^{2j}$ by a dummy adjacency edge, associating this pair to the dummy segment $\varphi ^{j_\circ } _{\mathbb {B}} \in U_{\mathbb {B}}^\varphi$.
($$4_\thickapprox$$)
For each edge $AB \in {\widehat{F}}$, let $A \in {\widehat{U}}_{\mathbb {A}}$ and $B \in {\widehat{U}}_{\mathbb {B}}$ be associated, respectively, to the cap vertices $\theta _{{\mathbb {A}}}^{2i-1},\theta _{{\mathbb {A}}}^{2i} \in {\hat{\theta }}({\mathbb {A}})$ and $\theta _{{\mathbb {B}}}^{2j-1}, \theta _{{\mathbb {B}}}^{2j} \in {\hat{\theta }}({\mathbb {B}})$. Connect the cap vertices with cap edges in the two crosswise possibilities: $\{\theta _{{\mathbb {A}}}^{2i-1},\theta _{{\mathbb {B}}}^{2j-1}\}$, $\{\theta _{{\mathbb {A}}}^{2i},\theta _{{\mathbb {B}}}^{2j}\}$ (1st of *A* to 1st of *B*, 2nd of *A* to 2nd of *B*), and also $\{\theta _{{\mathbb {A}}}^{2i-1},\theta _{{\mathbb {B}}}^{2j}\}$, $\{\theta _{{\mathbb {A}}}^{2i},\theta _{{\mathbb {B}}}^{2j-1}\}$ (1st of *A* to 2nd of *B*, 2nd of *A* to 1st of *B*). [Simple optimization: if the edge *AB* is a complete component in ${\widehat{\mathcal {C}}}({\mathbb {A}},{\mathbb {B}})$ (that is, both vertices *A* and *B* have degree one) and, moreover, either *A* or *B* is a dummy segment, then, since both ends of a dummy segment are “equivalent”, we simply remove one of the two crosswise connections of cap vertices described above (arbitrarily chosen).] The set of all cap edges is denoted by $E_{\theta }$.

Denote by ${\mathfrak {P}}_{\thickapprox }$ the set of all possible capping-sets (perfect matchings) between the vertices of ${\hat{\theta }}({\mathbb {A}})$ and ${\hat{\theta }}({\mathbb {B}})$. The optimization problem over $\theta _\thickapprox (F\!F\!R({\mathbb {A}},{\mathbb {B}},\mathcal {S}))$ is defined as

$$\begin{aligned} \textsc {GenDiFF}\!\!\thickapprox\!\!({\mathbb {A}}, {\mathbb {B}}, \mathcal {S}) = \displaystyle \min _{{\mathcal {L}}\in {\mathfrak {S}}, P\in {\mathfrak {P}}_{\thickapprox }}\{\textrm{wd} _\textsc {dcj}^\textsc {id}({\widehat{D}}[{\mathcal {L}},P])\}\,\text {.} \end{aligned}$$

Assuming that a sibling set ${\mathcal {L}}_\thickapprox$ (together with one of its best heuristic capping-sets) gives the optimal solution for $\textsc {GenDiFF}\!\!\thickapprox\!\!({\mathbb {A}}, {\mathbb {B}}, \mathcal {S})$, a best heuristic ortholog-set is $\textsc {OrthoFF}\!\!\thickapprox\!\!({\mathbb {A}}, {\mathbb {B}}, \mathcal {S}) = {\mathcal {O}}_{{\mathcal {L}}_\thickapprox }$. Both problems $\textsc {GenDiFF}\!\!\thickapprox$ and $\textsc {OrthoFF}\!\!\thickapprox$ can also be solved with the ILP $\text{FF-DCJ-I}\textsc {ndel}$, shown in Additional file 1: Section (A).

### Search space compared to optimal capping

If the threshold $\tau$ is similar to the numbers $\kappa ({\mathbb {A}})$ and $\kappa ({\mathbb {B}})$ of linear segments in each genome, and in the unlike situation where all linear segments from ${\mathbb {A}}$ are connected to all linear segments from ${\mathbb {B}}$ in ${\widehat{\mathcal {C}}}$, the heuristic capping induced by ${\widehat{\mathcal {C}}}$ may be as large as the optimal capping.

Therefore, $\tau$ is thought to be smaller than $\kappa ({\mathbb {A}})$ and $\kappa ({\mathbb {B}})$, effectively reducing the number of capping-sets. We could not yet estimate this reduction as a function of $\tau$, though. As our experimental results with real genomes show (details below, in the next section), with a small $\tau$ the heuristic capping leans to a considerably smaller number of capping-sets in practice.

### Integration of pairwise heuristic ortholog-sets into gene families

**Fig. 5:**
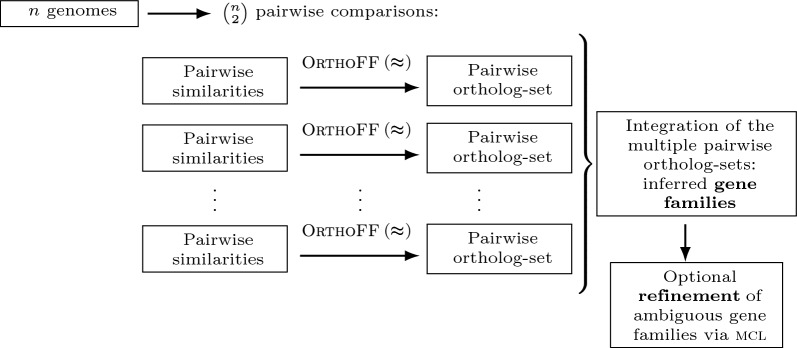
The pipeline of our approach is straightforward: our gene families are the connected components of the *n*-partite graph derived by the integration of the computed ortholog-sets. The resulting ambiguous families can be optionally refined with the help of $\textsc {mcl}$

**Fig. 6:**
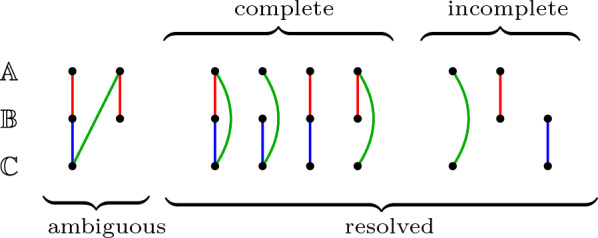
Types of families given by the integration of three ortholog-sets into a 3-partite graph

The ILP $\text{FF-DCJ-I}\textsc {ndel}$ solving $\textsc {OrthoFF}\!\!\thickapprox$ (with heuristic capping) is the core of a new version of our tool, called $\text{O} \textsc{rtho} \text{FFGC}\!\!\thickapprox$, for inferring gene families across several species, as illustrated in Fig. 5. Recall that each family is a connected component of the *n*-partite graph obtained by the simple integration of the computed pairwise ortholog-sets. An *ambiguous* family corresponds to a connected component of the *n*-partite graph that has more than one gene from the same genome. Otherwise we have a *resolved* family, which can be either *complete*, when it contains one gene per genome, or *incomplete* otherwise. Figure 6 illustrates these types of families in a 3-partite graph.

### Refinement of ambiguous gene families via $\textsc {mcl}$

Both pipelines $\text{O} \textsc{rtho} \text{FFGC}$ and $\text{O} \textsc{rtho} \text{FFGC}\!\!\thickapprox$ may produce a small number of large ambiguous families with low connectivity that can benefit from a further refinement (e.g. by removing bridges or making edge cuts in areas with small edge-connectivity) in order to produce more cohesive families. In an optional extra step, as shown in Fig. 5, our pipeline refines ambiguous families with the help of mcl [19], a fast and scalable clustering algorithm based on simulation of stochastic flow in graphs.

## Implementation and experiments

The pipeline $\text{O} \textsc{rtho} \text{FFGC}$ (with optimal capping) was previously integrated into the $\textsc {FFGC}$ workflow [12, 22], which includes the pre-computation of gene similarities, allowing therefore the automatic generation of families directly from the genome data. We implemented the new pipeline $\text{O} \textsc{rtho} \text{FFGC}\!\!\thickapprox$ (with heuristic capping) as another extension of the same workflow. The optional $\textsc {mcl}$ step for refining ambiguous families is integrated to both $\text{O} \textsc{rtho} \text{FFGC}$ and $\text{O} \textsc{rtho} \text{FFGC}\!\!\thickapprox$. The implementation and its documentation can be downloaded from our GitLab server at https://gitlab.ub.uni-bielefeld.de/gi/FFGC or as a Conda package at https://anaconda.org/bioconda/ffgc. (Note that mcl is an external dependency, automatically installed only if the download is obtained from Conda.)

An important recent modification of our pipeline is that now it performs pairwise similarity computations by default via $\textsc {diamond}$ [23] and no longer via $\textsc {blast}$ [24]. This change and some further optimizations improved greatly the running times of $\text{O} \textsc{rtho} \text{FFGC}\!\!\thickapprox$, that are now closer to the fastest alternative tools. Suprisingly, adopting $\textsc {diamond}$ also produced a small improvement of the quality of our gene families, compared to the version previously published [18]. The current values for quality and running times can be found in the end of this section.

### Alternative inference tools used for comparison purposes

$\text{P} \textsc {rotein} \text{O} \textsc {rtho}$ and $\textsc {Poff}$.   $\text{P} \textsc {rotein} \text{O} \textsc {rtho}$ [16] is a fast tool that clusters genes to find significant orthologous groups, based on a heuristic of reciprocal best hits of the corresponding sequence similarities. Its $\textsc {Poff}$ extension [17] takes into account gene adjacencies as an additional criterion for the discrimination of orthologs.

$\textsc {Oma}$.   Based on sequence similarities and on phylogeny, $\textsc {Oma}$ [25] is the underlying tool of the homonym online orthology browser. The standalone tool allows custom genomes to be compared to infer orthologous groups. Two types of families are reported: *hierarchical orthologous groups* ($\textsc {OmaHOGs}$), which may include ambiguous families, and *resolved groups* ($\textsc {OmaGroups}$).

### Gene similarities in $\text{O} \textsc{rtho} \text{FFGC}$/${\textsc {OrthoFF}\textsc {GC}}\!\!\thickapprox$

The computation of gene similarities is done via $\textsc {FFGC}$ and is described as follows. For any given pair of genes *x* and *y*, the software diamond is used for computing the value $\textsc {bitscr}(x\rightarrow y)$, that corresponds to the bitscore of gene *x* *with respect to* gene *y*. If gene *x* is in genome ${\mathbb {A}}$ and gene *y* is in genome ${\mathbb {B}}$, both $\textsc {bitscr}(x\rightarrow y)$ and $\textsc {bitscr}(y\rightarrow x)$ must be taken into consideration for calculating the similarity $\sigma (x,y)$, that is equivalent to their *relative reciprocal score* [26]:

$$\begin{aligned} \sigma (x,y)=\frac{\textsc {bitscr}(x\rightarrow y) + \textsc {bitscr}(y\rightarrow x)}{\textsc {bitscr}(x\rightarrow x)+\textsc {bitscr}(y\rightarrow y)}. \end{aligned}$$

Negligible similarities are then identified and discarded by the filter $\digamma$, by requiring that (1) for a first given threshold $\digamma _{\epsilon } \in (0, 1]$, $\sigma (x, y) \ge \digamma _{\epsilon }$ (*absolute filter*); and (2) for a second given threshold $\digamma _{t} \in (0, 1]$, the value $\textsc {bitscr}(x\rightarrow y)$ must be at least a $\digamma _{t}$-fraction of the highest bitscore of *x* with respect to any gene in ${\mathbb {B}}$ and the value $\textsc {bitscr}(y\rightarrow x)$ must be at least a $\digamma _{t}$-fraction of the highest bitscore of *y* with respect to any gene in ${\mathbb {A}}$ (*relative minimum reciprocal similarity for additional hits*, which was developed and used in the alternative tools $\text{P} \textsc {rotein} \text{O} \textsc {rtho}$ [16] and $\textsc {Poff}$ [17]). Once these conditions are fulfilled, the edge *xy* is added to the gene similarity graph with score $\sigma (xy)=\sigma (x,y)$. The adopted values are $\digamma _{\epsilon }=0.1$ and $\digamma _{t}=0.8$, the latter also being adopted for $\text{P} \textsc {rotein} \text{O} \textsc {rtho}$ and $\textsc {Poff}$ whenever these tools were used in our experiments.

The default values of the other parameters for the pre-computation of gene similarities via $\textsc {FFGC}$ were kept, except for one of them: the minimum number of genomes for which each gene must share some similarity in is set to 1, otherwise genes not similar to any other gene, which should be still considered in indels, would not appear in the chromosomal gene order.

### Computational environment and additional parameters of $\text{O} \textsc{rtho} \text{FFGC}\!\!\thickapprox$

We ran experiments in a 2.7GHz multi-core machine. Whenever possible, tasks ran using 8 cores. As an ILP solver, we used Gurobi.

For the post-processing refinement of ambiguous families in $\text{O} \textsc{rtho} \text{FFGC}\!\!\thickapprox$, we used the default parameters of mcl with a conservative “inflation” value of 1.4 suggested in its manual (https://micans.org/mcl).

In the following we will describe our experiments, based on genome assemblies fetched from NCBI. We performed two distinct comparisons. First, based on a set of five completely assembled primate genomes, we compared $\text{O} \textsc{rtho} \text{FFGC}\!\!\thickapprox$ families (inferred with the heuristic capping) to $\text{O} \textsc{rtho} \text{FFGC}$ families (inferred with the optimal capping). Then, based on the set of 11 *Drosophilas* including partially assembled genomes, we compared $\text{O} \textsc{rtho} \text{FFGC}\!\!\thickapprox$ families to the gene families inferred by other tools, using $\text{F} \textsc {ly} \text{B} \textsc{ase}$ families as reference.

### Analysis of completely assembled primate genomes: comparing $\text{O} \textsc{rtho} \text{FFGC}\!\!\thickapprox$ to $\text{O} \textsc{rtho} \text{FFGC}$

The goal of this experiment is to evaluate the impact of the heuristic capping on running times and on the quality of results, with respect to the optimal capping in a dataset of large genomes. We compared families inferred by $\text{O} \textsc{rtho} \text{FFGC}$ and $\text{O} \textsc{rtho} \text{FFGC}\!\!\thickapprox$, considering the following five primate genomes: bonobo (*P. paniscus*), chimpanzee (*P. troglodytes*), gorilla (*G. gorilla*), human (*H. sapiens*) and orangutan (*P. abelii*). Those genomes comprise roughly $20{,}000 \sim 22{,}000$ genes distributed in 25 chromosomes, except for the human genome that has 24 chromosomes. The average number of edges (similarities) for each vertex (gene) in the gene similarity graphs is 1.09, totaling 429268 vertices and 234297 edges. Considering only the genes with multiple similarities, the average degree is 2.93.

For the capping heuristic in $\text{O} \textsc{rtho} \text{FFGC}\!\!\thickapprox$, we set $\epsilon =0.1$ and $\tau = 2$, a choice that reduces significantly the number of capping sets, but still gives some options to the ILP solver. With these choices, the largest number of edges in ${\widehat{\mathcal {C}}}({\mathbb {A}},{\mathbb {B}})$ was for the comparison of gorilla and human. In this case, ${\widehat{\mathcal {C}}}$ has 27 edges distributed among 21 components with 2 vertices, and 2 components with 4 vertices (including 1 dummy segment). That corresponds to at most $\sim 1.1 \times 10^6$ capping-sets in $\theta _\thickapprox$, while the pairwise comparison with the optimal $\theta _\star$ has 50! capping-sets.

This significant reduction of the search space enabled 7 out of 10 pairwise comparisons with the heuristic capping to finish within the time limit of 60 min, in contrast to the lengthy comparisons with the optimal capping, that were limited to 1440 min (24 h). The results are shown in Table 1, where we can also see that the size of the ortholog-sets ${\mathcal {O}}$, the number of DCJ-indel operations $\textrm{d} _\textsc {dcj} ^\textsc {id}$, and the weighted rearrangement distances $\textrm{wd} _\textsc {dcj}^\textsc {id}$ of the two approaches are almost identical for all pairwise comparisons. While 21090 families were inferred using $\text{O} \textsc{rtho} \text{FFGC}$, 21101 were inferred using $\text{O} \textsc{rtho} \text{FFGC}\!\!\thickapprox$, and 99.2% of those families are the same, showing that moving from the optimal capping to the heuristic capping of the family-free relational diagram had very little impact on the gene family inference. The total running time of $\text{O} \textsc{rtho} \text{FFGC}\!\!\thickapprox$ was of about 321 min (of which 306 min were spent by the pairwise ILP computations). The numbers of classified genes and of inferred families are displayed in Table 2.

**Table 1:**
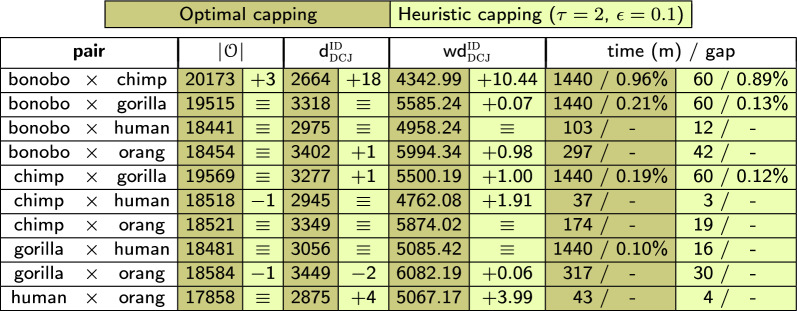
ILP running times, DCJ-indel distances and weighted DCJ-indel distances with optimal and heuristic cappings for the pairwise comparisons of primate genomes

This experiment accomplished its goal, by showing that the heuristic capping can have the same quality of the optimal capping, while having substantial impact in speeding up the ILP computations. Indeed, the time required to analyze the dataset of five primates with $\text{O} \textsc{rtho} \text{FFGC}\!\!\thickapprox$ was quite reasonable. However it was still considerably longer than the fastest alternative tool: the total running time of $\text{P} \textsc {rotein} \text{O} \textsc {rtho}$ for the same dataset was of about 19 min only, with the corresponding numbers of classified genes and inferred families being also displayed in Table 2. It is worth mentioning that in $\text{O} \textsc{rtho} \text{FFGC}\!\!\thickapprox$ a resolved family has the additional confidence of resulting from independently computed pairwise ortholog-sets. Therefore it is remarkable that $\text{O} \textsc{rtho} \text{FFGC}\!\!\thickapprox$ could identify a significantly higher number of resolved complete families, including almost all resolved complete families also inferred by $\text{P} \textsc {rotein} \text{O} \textsc {rtho}$.

**Table 2:**
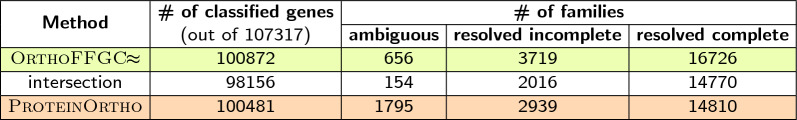
Numbers of classified genes and families inferred by $\text{O} \textsc{rtho} \text{FFGC}\!\!\thickapprox$ and $\text{P} \textsc {rotein} \text{O} \textsc {rtho}$ for the dataset of five primates

### Analysis of partially assembled *Drosophila* (fruit flies) genomes

The $\text{F} \textsc {ly} \text{B} \textsc{ase}$ consortium (https://flybase.org) sequenced, assembled and annotated the genomes of 12 *Drosophilas* with $\sim 12{,}000$–16,000 protein-coding genes (for genes with multiple transcripts, we kept only the longest), however only 11 of those genomes are available on NCBI together with the complete annotation: *D. ananassae*, *D. erecta*, *D. grimshawi*, *D. melanogaster*, *D. mojavensis*, *D. persimilis*, *D. sechellia*, *D. simulans*, *D. virilis*, *D. willistoni* and *D. yakuba*.

In the family-free analysis, the total number of vertices and edges in the gene similarity graphs are 1514930 and 633521, respectively, with average degrees of 0.83 considering all vertices, and 2.22 looking only at those genes with multiple similarities. For the capping heuristic in $\text{O} \textsc{rtho} \text{FFGC}\!\!\thickapprox$, we again set $\epsilon =0.1$ and $\tau = 2$.

#### Average numbers of cap edges and capping-sets in $\theta _\star$ and in $\theta _\thickapprox$

The analyzed *Drosophila* genomes have 507 contigs on average, therefore each optimally capped family-free relational diagram has $1{,}014 \times 1{,}014 = 1{,}028{,}196$ cap edges and an unfeasible total of 1, 014! capping-sets on average.

In contrast, considering the perfect shared-content graphs for all pairwise *Drosophila* comparisons, 99.7% of the components in those graphs have only 1 linear segment in each part of the graph. In the remaining 0.3%, 80% have 7 or fewer linear segments in each part, with the largest component having 76 linear segments in each part. The perfect shared-content graphs have an average of 1,419 edges. For that number of edges, each heuristically capped family-free relational diagram has 5,676 cap edges on average. As the exact number of perfect matchings in arbitrary graphs is not trivial to estimate, we computed an upper limit for the average number of distinct capping-sets by the Bregman-Cinc inequality [27] of the permanent of a squared matrix: $\sim 45!$, with median $\sim 18!$.

#### Benchmark for our experiments

Reference families were obtained directly from $\text{F} \textsc {ly} \text{B} \textsc{ase}$ (https://flybase.org). Since the set of genes classified in $\text{F} \textsc {ly} \text{B} \textsc{ase}$ is slightly different from the set of genes present with their coding sequences in database files, we filtered out a small portion ($\sim 7\%$) of genes in $\text{F} \textsc {ly} \text{B} \textsc{ase}$ families so that only those present in the NCBI databases with their coding sequences were kept. Prior to any comparison of inferred families to $\text{F} \textsc {ly} \text{B} \textsc{ase}$ families, we also filtered out from the inferred families genes not present in $\text{F} \textsc {ly} \text{B} \textsc{ase}$ families.

#### Comparing $\text{O} \textsc{rtho} \text{FFGC}\!\!\thickapprox$ to $\text{P} \textsc {rotein} \text{O} \textsc {rtho}$, $\textsc {Poff}$ and $\textsc {Oma}$

We analyzed the dataset of 11 *Drosophila* genomes for comparing the performance of $\text{O} \textsc{rtho} \text{FFGC}\!\!\thickapprox$ against $\text{P} \textsc {rotein} \text{O} \textsc {rtho}$, $\textsc {Poff}$ and $\textsc {Oma}$, providing as additional input to the latter the phylogenetic tree (obtained from FlyBase) of the same 11 *Drosophilas*. Unlocalized contigs were not filtered out, resulting in genomes with 11 to 1041 linear segments.

##### Quality of inferred families

The numbers of families in $\text{F} \textsc {ly} \text{B} \textsc{ase}$ and those inferred by $\textsc {OmaHOGs}$, $\textsc {OmaGroups}$, $\text{P} \textsc {rotein} \text{O} \textsc {rtho}$, $\textsc {Poff}$ and $\text{O} \textsc{rtho} \text{FFGC}\!\!\thickapprox$ are all bigger than 12,000. In order to have a hint on the quality of results, we focus on the intersections with $\text{F} \textsc {ly} \text{B} \textsc{ase}$ families and on *precision* and *recall* values computed for all methods.

**Table 3:**
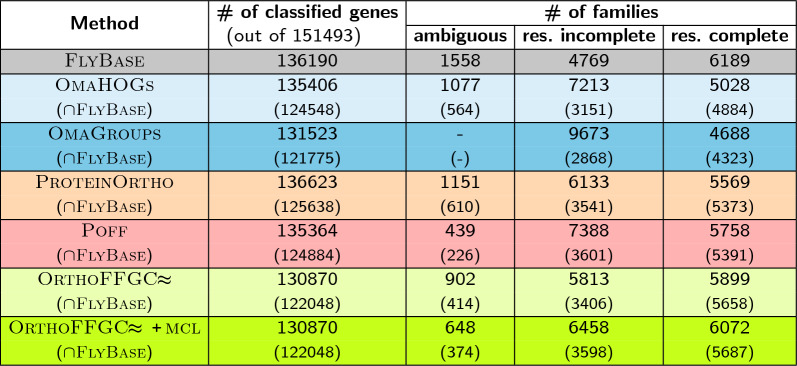
Numbers of classified genes and families inferred by the different methods for the dataset of 11 Drosophilas

**Fig. 7:**
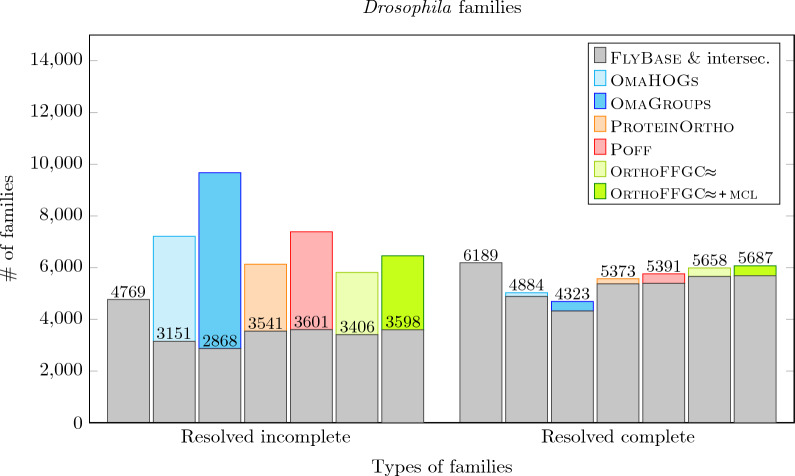
The numbers of resolved incomplete and complete families in $\text{F} \textsc {ly} \text{B} \textsc{ase}$, followed by the numbers of families inferred by $\textsc {OmaHOGs}$, $\textsc {OmaGroups}$, $\text{P} \textsc {rotein} \text{O} \textsc {rtho}$, $\textsc {Poff}$ and $\text{O} \textsc{rtho} \text{FFGC}\!\!\thickapprox$ (without and with $\textsc {mcl}$ refinement). The lower part of each bar represents the intersection between the inferred sets and $\text{F} \textsc {ly} \text{B} \textsc{ase}$. (For resolved complete families, the numbers of families in the intersections are shown on the top of bars)

The numbers of classified genes and families inferred by all methods, together with the respective intersections with $\text{F} \textsc {ly} \text{B} \textsc{ase}$ are shown in Table 3. Figure 7 shows the comparative picture focusing on the numbers of resolved incomplete and complete families for all methods.

We also counted the numbers of pairwise gene homologies that are classified as *true positive* (TP), *false positive* (FP) and *false negative* (FN) as follows. First, denote a subset of size two by *2-subset*. Now let ${\mathcal {H}}_\textsc {fly}$ be the set composed of 2-subsets of all $\text{F} \textsc {ly} \text{B} \textsc{ase}$ families, and, for any considered set of families *X*, let ${\mathcal {H}}_X$ be the set composed of 2-subsets of all families in *X*. Then TP of *X* is the size of ${\mathcal {H}}_\textsc {fly}\! \cap \!{\mathcal {H}}_X$, FN of *X* is the size of ${\mathcal {H}}_\textsc {fly}\!\setminus \!{\mathcal {H}}_X$ and FP of *X* is the size of ${\mathcal {H}}_X\!\setminus \!{\mathcal {H}}_\textsc {fly}$. Based on that we computed the values of *precision* $\left( \frac{\text {TP}}{\text {TP}+\text {FP}}\right)$ and *recall* $\left( \frac{\text {TP}}{\text {TP}+\text {FN}}\right)$ for the sets of families inferred by the considered methods.

**Fig. 8:**
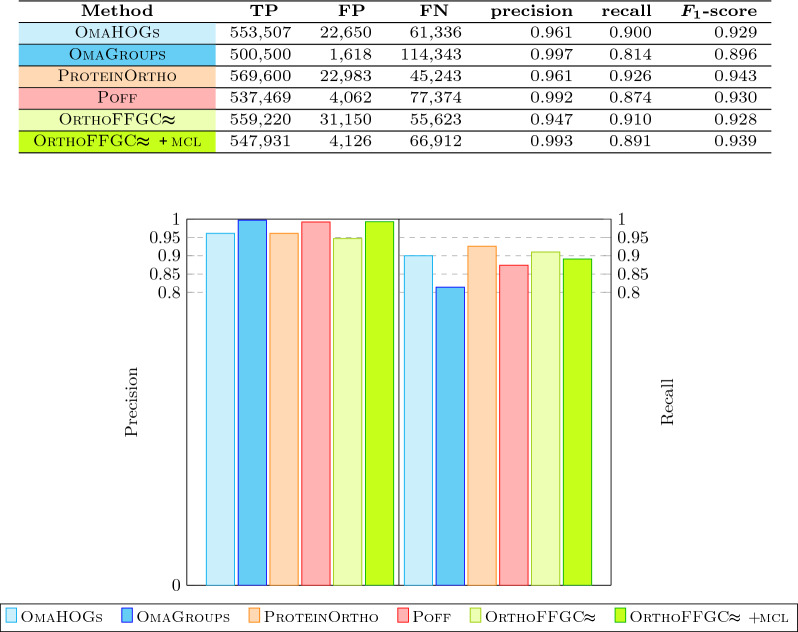
Precision $\left( \frac{\text {TP}}{\text {TP}+\text {FP}}\right)$, recall $\left( \frac{\text {TP}}{\text {TP}+\text {FN}}\right)$ and their harmonic mean $F_1$-score for $\textsc {OmaHOGs}$, $\textsc {OmaGroups}$, $\text{P} \textsc {rotein} \text{O} \textsc {rtho}$, $\textsc {Poff}$ and $\text{O} \textsc{rtho} \text{FFGC}\!\!\thickapprox$ (without and with $\textsc {mcl}$ refinement), based on the dataset with eleven *Drosophilas*

The results (Fig. 8) show that, while all methods performed quite well, our tool $\text{O} \textsc{rtho} \text{FFGC}\!\!\thickapprox$ had the lowest precision. However, when the optional $\textsc {mcl}$ refinement step is enabled in our pipeline, its overall results were improved, with an increase of the precision that is bigger than the corresponding decrease of the recall, reflected on the increase of the $F_1$-score.

##### Running times

We compiled the running times of the four methods in Table 4. The (preprocessing) step 1 of computing the pairwise sequence similarities is required by all methods, but, while $\textsc {Oma}$ has an internal implementation of the Smith-Waterman algorithm, the other methods used diamond [23], which is the fastest known tool for accomplishing that task.

**Table 4:**
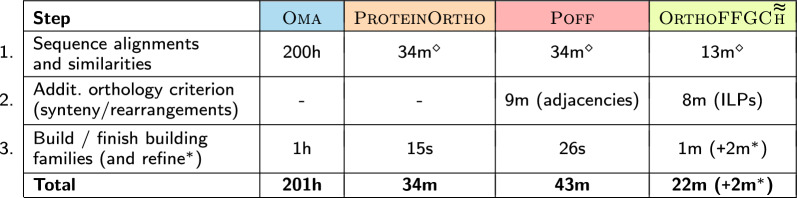
Running times for computing *Drosophila* families

$$^\diamond$$ Computed with $$\textsc {diamond}$$

$^*$ The optional additional $\textsc {mcl}$ refinement step on $\text{O} \textsc{rtho} \text{FFGC}\!\!\thickapprox$ takes 2 extra minutes

Having at hand the sequence similarities, $\text{P} \textsc {rotein} \text{O} \textsc {rtho}$ and $\textsc {Oma}$ build families taking into consideration alignments and similarities. Additionally, $\textsc {Oma}$ takes into consideration the provided phylogenetic tree of the 11 *Drosophilas*. The running times of these procedures are given in step 3. For the other methods, we separated in step 2 core procedures that use additional criteria to find pairwise orthologs: $\textsc {Poff}$ does it via the analysis of synteny by means of conserved gene adjacencies, while $\text{O} \textsc{rtho} \text{FFGC}\!\!\thickapprox$ generates and solves the ILPs for obtaining pairwise $\textsc {OrthoFF}\!\!\thickapprox$ gene orthologies.

Adopting $\textsc {diamond}$ for similarity computations instead of $\textsc {blast}$ was an important recent modification of our pipeline. This change and the use of more restrictive parameters for the heuristic capping improved greatly the running times of our method compared to its previous version [18]. With these improvements the running times of $\text{O} \textsc{rtho} \text{FFGC}\!\!\thickapprox$ are now the fastest among the compared tools for the *Drosophila* dataset.

## Conclusions and discussion

We devised and implemented a heuristic capping for improving our recently developed pipeline $\text{O} \textsc{rtho} \text{FFGC}$ [13] for inferring gene families based on genome rearrangements. In $\text{O} \textsc{rtho} \text{FFGC}$ we adopted an optimal capping including all connections between the ends of linear segments to allow all possible $(2p_*)!$ capping-sets in the input of the ILP $\text{FF-DCJ-I}\textsc {ndel}$ that infers the $\text{O} \textsc{rtho} \text{FF}$ pairwise orthologs. However, due to the heavy optimal capping, $\text{FF-DCJ-I}\textsc {ndel}$ can hardly converge when analyzing larger genomes (such as mammals) and fails in handling a pair of genomes if one or both of them (with at least the dimension of a fruit fly genome) are distributed in a hundred contigs.

In contrast, the new pipeline $\text{O} \textsc{rtho} \text{FFGC}\!\!\thickapprox$ adopts a lighter heuristic capping including connections only between linear segments that share gene content. This leads to a much smaller number of capping-sets in the input of the same $\text{FF-DCJ-I}\textsc {ndel}$ that here infers $\textsc {OrthoFF}\!\!\thickapprox$ pairwise orthologs. Despite the use of a heuristic capping, our evaluation showed that the quality of the orthologies inferred by $\text{O} \textsc{rtho} \text{FFGC}\!\!\thickapprox$ was very good.

A first evaluation on a dataset of five completely assembled primate genomes was done by comparing the families inferred by the previous workflow $\text{O} \textsc{rtho} \text{FFGC}$ with the new $\text{O} \textsc{rtho} \text{FFGC}\!\!\thickapprox$. The results showed that the gene families inferred by the two pipelines are virtually the same. Therefore, in practice, the heuristic capping did not have a negative impact on the inferred gene families, essentially preserving the original (optimal) orthology relations.

A second evaluation was done on a dataset of 11 *Drosophila* genomes, including partially assembled genomes distributed in several contigs, by adopting the gene families curated by the $\text{F} \textsc {ly} \text{B} \textsc{ase}$ consortium as a benchmark. In this experiment we compared $\text{O} \textsc{rtho} \text{FFGC}\!\!\thickapprox$ to other genome-scale methods, namely $\textsc {Oma}$, $\text{P} \textsc {rotein} \text{O} \textsc {rtho}$ and $\textsc {Poff}$. The running times of $\text{O} \textsc{rtho} \text{FFGC}\!\!\thickapprox$ for the *Drosophila* dataset are better than the fastest alternative tools $\text{P} \textsc {rotein} \text{O} \textsc {rtho}$ and $\textsc {Poff}$, showing that our implementation is very efficient, despite the pairwise ILP computations. Furthermore, our results showed that $\text{O} \textsc{rtho} \text{FFGC}\!\!\thickapprox$ was able to infer only 3 resolved families less than $\text{P} \textsc {rotein} \text{O} \textsc {rtho}$ and had the highest number of resolved complete families in common with $\text{F} \textsc {ly} \text{B} \textsc{ase}$, and these intersections were improved after the refinement of ambiguous families with $\textsc {mcl}$. Concerning the analysis of pairwise gene orthologies derived from the inferred families, all tools had a very good performance. After the $\textsc {mcl}$ refinement our tool reached the second best $F_1$-score, with a difference of only 0.004 to the best $F_1$-score achieved by $\text{P} \textsc {rotein} \text{O} \textsc {rtho}$.

The bottleneck of our pipeline is still the ILP pairwise computations that, despite the gain of heuristic capping, solve instances of an NP-hard problem. However, the heuristic capping allows to efficiently analyze large genomes such as mammals and, at least for genomes with the dimension of a fruit fly genome, $\text{O} \textsc{rtho} \text{FFGC}\!\!\thickapprox$ lifts the limitation of requiring chromosome-level assembled genomes, expanding to a great extent its applicability.

### Supplementary Information

**Additional file 1.** Online supplemental material

## Data Availability

See https://gitlab.ub.uni-bielefeld.de/gi/FFGC or https://anaconda.org/bioconda/ffgc.

## Footnotes

1 Another attempt called $\textsc{msoar}$ (Shi, Zhang and Jiang, BMC Bioinformatics 11:10, 2010) was made before our studies, the differences being that $\textsc{msoar}$ first infers gene families based on similarities and then computes a matching based on a heuristic including structural rearrangements and tandem duplications, while $\text{FF-DCJ-I}\textsc{ndel}$ takes similarities and rearrangements simultaneously into account for inferring an optimal matching, in a rearrangement model including DCJ and mimicking all content modifications with insertions and deletions of DNA segments. The tool $\textsc{msoar}$ was not maintained and is no longer operational, therefore we could never compare its performance to $\text{FF-DCJ-I}\textsc{ndel}$.

2 Our method was originally called $\textsc {DiffMGC}$ [13], here renamed to $\text{O} \textsc{rtho} \text{FFGC}$.

3 There can be several co-optimal capping-sets for the same decomposition $D[{\mathcal {L}}]$ and each optimal capping-set links up to 4 cycles of $D[{\mathcal {L}}]$ into a single cycle [8].
