# Supplementary material for: Efficient gene orthology inference via large-scale rearrangements
Source: Algorithms Mol Biol. 2023 Sep 28;18:14. doi: 10.1186/s13015-023-00238-y (PMC10540461; doi:10.1186/s13015-023-00238-y)
Supplement: Supplementary file 1 — Additional file 1. Online supplemental material [file 13015_2023_238_MOESM1_ESM.pdf]

Additional File 1 - Supplemental material of: **Efficient gene orthology inference via large-scale rearrangements**

Diego P. Rubert[1][2] and Marília D. V. Braga[2]

(A) ILP formulation for family-free DCJ-indel

The ILP, shown in Algorithm A1, was developed in our previous work [12], but there it only considered an optimal capping of a family-free relational graph $\mathit{FFR}(\mathbb{A},\mathbb{B},\mathcal{S})$. It is an adaptation of the ILP for computing the DCJ-indel distance of family-based natural genomes, by Bohnenkämper *et al.* [8], which is itself an extension of the ILP for computing the DCJ distance of family-based balanced genomes, by Shao *et al.* [7].

Algorithm A1 FF-DCJ-INDEL: ILP for computing the best DCJ-indel weighted cost

Input: A family-free relational graph $\mathit{FFR}(\mathbb{A},\mathbb{B},\mathcal{S})$ with a valid capping $\theta_\star$ or $\theta_\approx$

$$\min \quad p + \sum_{e \in E_\gamma} x_e - \sum_{1 \le i \le |V|} z_i + \sum_{k \in K} s_k + \frac{1}{2}\sum_{e \in E} t_e - \frac{1}{2}\sum_{e \in E_\gamma} w_e x_e + \sum_{e \in E_{\mathsf{id}}} w_e x_e$$

$$\begin{array}{llcll}
\text{s. t.} & x_e & = & 1 & \forall\, e \in E^{\mathbb{A}}_{\mathsf{adj}} \cup E^{\mathbb{B}}_{\mathsf{adj}} \quad \text{(C.01)} \\
& \sum_{uv \in E} x_{uv} & = & 2 & \forall\, u \in V \quad \text{(C.02)} \\
& x_e & = & x_d & \forall\, e, d \in E_\gamma,\ e, d \text{ are siblings} \quad \text{(C.03)} \\
& y_i & \le & y_j + i(1 - x_{v_i v_j}) & \multirow{2}{*}{} \forall\, v_i v_j \in E \quad \text{(C.04)} \\
& y_j & \le & y_i + j(1 - x_{v_i v_j}) & \\
& y_i & \le & i(1 - x_{v_i v_j}) & \forall\, v_i v_j \in E^{\mathbb{A}}_{\mathsf{id}} \cup E^{\mathbb{B}}_{\mathsf{id}} \quad \text{(C.05)} \\
& y_j & \le & j(1 - x_{v_i v_j}) & \\
& i z_i & \le & y_i & \forall\, 1 \le i \le |V| \quad \text{(C.06)} \\
& r_v & \le & 1 - x_{uv} & \forall\, uv \in E^{\mathbb{A}}_{\mathsf{id}} \quad \text{(C.07)} \\
& r_{v'} & \ge & x_{u'v'} & \forall\, u'v' \in E^{\mathbb{B}}_{\mathsf{id}} \\
& t_{uv} & \ge & r_v - r_u - (1 - x_{uv}) & \forall\, uv \in E \quad \text{(C.08)} \\
& t_{uv} & \ge & r_u - r_v - (1 - x_{uv}) & \\
& \sum_{d \in E^{\mathbb{A}}_{\mathsf{id}},\ d \cap e \neq \emptyset} x_d - t_e & \ge & 0 & \forall\, e \in E^{\mathbb{A}}_{\mathsf{adj}} \quad \text{(C.09)} \\
& t_e & = & 0 & \forall\, e \in E \setminus E^{\mathbb{A}}_{\mathsf{adj}} \quad \text{(C.10)} \\
& \sum_{e \in E^{k}_{\mathsf{id}}} x_e - |k| & \le & s_k & \forall\, k \in K \quad \text{(C.11)} \\
\text{and} & x_e & \in & \{0,1\} & \forall\, e \in E \quad \text{(D.01)} \\
& 0 & \le & y_i \ \le\ i & \forall\, 1 \le i \le |V| \quad \text{(D.02)} \\
& z_i & \in & \{0,1\} & \forall\, 1 \le i \le |V| \quad \text{(D.03)} \\
& r_v & \in & \{0,1\} & \forall\, v \in V \quad \text{(D.04)} \\
& t_e & \in & \{0,1\} & \forall\, e \in E \quad \text{(D.05)} \\
& s_k & \in & \{0,1\} & \forall\, k \in K \quad \text{(D.06)} \\
& p & = & p_* \text{ (optimal capping) or } p_\approx \text{ (heuristic capping)} & \quad \text{(D.07)}
\end{array}$$

[1]Faculdade de Computação, Universidade Federal de Mato Grosso do Sul, Brazil
[2]Faculty of Technology and Center for Biotechnology (CeBiTec), Bielefeld University, Germany

ILP description

Given a valid capping θ (that here can be $\theta_\star$ or $\theta_\approx$), the general idea is searching for a sibling-set that, together with a capping-set, induces an optimal capped consistent decomposition of the capped diagram $\theta(\mathit{FFR}(\mathbb{A},\mathbb{B},\mathbb{S})) = (V,E)$, where:

- $V = V(\mathbb{A}) \cup V(\mathbb{B}) \cup \hat{\theta}(\mathbb{A}) \cup \hat{\theta}(\mathbb{B})$ (vertices representing gene extremities and cap extremities) and
- $E = E_\gamma \cup E_\theta \cup E^{\mathbb{A}}_{\text{adj}} \cup E^{\mathbb{B}}_{\text{adj}} \cup E^{\mathbb{A}}_{\text{id}} \cup E^{\mathbb{B}}_{\text{id}}$ (the set of edges comprises all disjoint sets of distinct edge types).
- Additionally, we define the set K that contains all circular chromosomes of both genomes $\mathbb{A}$ and $\mathbb{B}$. Without loss of generality, let k be a circular chromosome in genome $\mathbb{A}$. In the ILP we also refer to the set of edges $E^k_{\text{id}} \subseteq E^{\mathbb{A}}_{\text{id}}$.

The same ILP formulation (shown in Algorithm A1) computes

$$\text{either} \quad \textsc{GenDiFF}(\mathbb{A},\mathbb{B},\mathbb{S}) \quad \text{and} \quad \textsc{OrthoFF}(\mathbb{A},\mathbb{B},\mathbb{S}) \quad (\text{with } \theta_\star)$$

$$\text{or} \quad \textsc{GenDiFF}{\approx}(\mathbb{A},\mathbb{B},\mathbb{S}) \quad \text{and} \quad \textsc{OrthoFF}{\approx}(\mathbb{A},\mathbb{B},\mathbb{S}) \quad (\text{with } \theta_\approx).$$

A particular feature of this ILP when compared to those from [7] and [8] is that its search space is not restricted to maximal sibling-sets but includes all sibling-sets, of any size.

For capturing the properties required for computing the best DCJ-indel weighted cost, whose details can be found in [12, 13], the ILP (Algorithm A1) is distributed in three main parts:

1 Counting indel-free cycles (those without indel edges) makes up the first part, depicted in constraints (C.01)–(C.06), variables and domains (D.01)–(D.03).

2 The second part is for counting transitions (paths between an indel edge in $\mathbb{A}$ and an indel edge in $\mathbb{B}$), described in constraints (C.07)–(C.10), variables and domains (D.04)–(D.05).

3 The last part describes how to count the number of circular singletons (circular chromosomes exclusively composed of indel and adjacency edges) with constraint (C.11), variable and domain (D.06).

The **objective function** of our ILP minimizes the size of the sibling-set (that is twice the size of the ortholog-set), with sum over variables x_e, the number of circular singletons, calculated by the sum over variables s_k, half the overall number of transitions in indel-enclosing (non-singletons) cycles, calculated by the sum over variables t_e, and the weight of all indel edges in the decomposition, given by the sum over their weights $w_e x_e$ for all $e \in E_{\text{id}}$, while maximizing both the number of indel-free cycles, counted by the sum over variables z_i, and half of the weight of the sibling-set.

Note that the minimization is not affected by constant p that corresponds to p_* when the graph is optimally capped or to $p_\approx$ when the graph is heuristically capped.

Availability

The ILP can be downloaded from our GitLab server at `gitlab.ub.uni-bielefeld.de/gi/gen-diff` and is integrated to OrthoFFGC/OrthoFFGC≈ pipelines at `gitlab.ub.uni-bielefeld.de/gi/FFGC`.

References (original list from the manuscript)

1. Bergeron, A., Mixtacki, J., Stoye, J.: A unifying view of genome rearrangements. In: Proc. of WABI. Lecture Notes in Bioinformatics, vol. 4175, pp. 163–173 (2006)
2. Hannenhalli, S., Pevzner, P.A.: Transforming men into mice (polynomial algorithm for genomic distance problem). In: Proc. of FOCS, pp. 581–592 (1995)
3. Braga, M.D.V., Willing, E., Stoye, J.: Double cut and join with insertions and deletions. J Comput Biol **18**(9), 1167–1184 (2011)
4. Sankoff, D.: Genome rearrangement with gene families. Bioinformatics **15**(11), 909–917 (1999)
5. Bryant, D.: The complexity of calculating exemplar distances. In: Sankoff, D., Nadeau, J.H. (eds.) Comparative Genomics. Computational Biology Series, vol. 1, pp. 207–211. Kluver Academic Publishers, London (2000)
6. Angibaud, S., Fertin, G., Rusu, I., Thévenin, A., Vialette, S.: On the approximability of comparing genomes with duplicates. J Graph Algo App **13**(1), 19–53 (2009)
7. Shao, M., Lin, Y., Moret, B.: An exact algorithm to compute the double-cut-and-join distance for genomes with duplicate genes. J Comput Biol **22**(5), 425–435 (2015)
8. Bohnenkämper, L., Braga, M.D.V., Doerr, D., Stoye, J.: Computing the rearrangement distance of natural genomes. J Comput Biol **28**(4), 410–431 (2021)
9. Yancopoulos, S., Attie, O., Friedberg, R.: Efficient sorting of genomic permutations by translocation, inversion and block interchange. Bioinformatics **21**(16), 3340–3346 (2005)
10. Braga, M.D.V., Chauve, C., Doerr, D., Jahn, K., Stoye, J., Thévenin, A., Wittler, R.: The potential of family-free genome comparison. In: Chauve, C., El-Mabrouk, N., Tannier, E. (eds.) Models and Algorithms for Genome Evolution. Computational Biology Series, vol. 19, pp. 287–307. Springer, Berlin (2013). Chap. 13
11. Martinez, F.V., Feijao, P., Braga, M.D.V., Stoye, J.: On the family-free DCJ distance and similarity. Algorithms Mol Biol **13**(10) (2015)
12. Rubert, D.P., Martinez, F.V., Braga, M.D.V.: Natural Family-Free Genomic Distance. Algorithms Mol Biol **16**(4) (2021)
13. Rubert, D.P., Doerr, D., Braga, M.D.V.: The potential of family-free rearrangements towards gene orthology inference. J Bioinform Comput Biol **19**(6), 2140014 (2021)
14. Dessimoz, C., Cannarozzi, G., Gil, M., Margadant, D., Roth, A.C.J., Schneider, A., Gonnet, G.H.: OMA, a comprehensive, automated project for the identification of orthologs from complete genome data: introduction and first achievements. In: Proc. of RECOMB-CG. Lecture Notes in Bioinformatics, vol. 3678, pp. 61–72 (2005)
15. Roth, A.C.J., Gonnet, G.H., Dessimoz, C.: Algorithm of OMA for large-scale orthology inference. BMC Bioinform **9**(518) (2008)
16. Lechner, M., Findeiß, S., Steiner, L., Marz, M., Stadler, P.F., Prohaska, S.J.: Proteinortho: Detection of (co-)orthologs in large-scale analysis. BMC Bioinform **12**(124) (2011)
17. Lechner, M., Hernandez-Rosales, M., Doerr, D., Wieseke, N., Thévenin, A., Stoye, J., Hartmann, R.K., Prohaska, S.J., Stadler, P.F.: Orthology detection combining clustering and synteny for very large datasets. PLoS One **9**(8:e105015) (2014)
18. Rubert, D.P., Braga, M.D.V.: Gene Orthology Inference via Large-Scale Rearrangements for Partially Assembled Genomes. In: Proc. of WABI. Leibniz International Proceedings in Informatics (LIPIcs), vol. 242, no. 24, pp. 1–22 (2022)
19. van Dongen, S.: Graph clustering via a discrete uncoupling process. SIAM Journal on Matrix Analysis and Applications **30**(1), 121–141 (2008)
20. Hall, P.: On representatives of subsets. Journal of the London Mathematical Society **s1-10**(1), 26–30 (1935)
21. Tassa, T.: Finding all maximally-matchable edges in a bipartite graph. Theoretical Computer Science **423**, 50–58 (2012)
22. Doerr, D., Feijão, P., Stoye, J.: Family-free genome comparison. In: Setubal, J.C., Stoye, J., Stadler, P.F. (eds.) Comparative Genomics: Methods and Protocols. Methods in Molecular Biology, vol. 1704, pp. 331–342. Springer, New York (2018)
23. Buchfink, B., Xie, C., Huson, D.H.: Fast and sensitive protein alignment using DIAMOND. Nat Methods **12**, 59–60 (2015)
24. Altschul, S.F., Gish, W., Miller, W., Myers, E.W., Lipman, D.J.: Basic local alignment search tool. J Mol Biol **215**(3), 403–410 (1990)
25. Altenhoff, A.M., Levy, J., Zarowiecki, M., Tomiczek, B., Vesztrocy, A.W., Dalquen, D.A., Müller, S., Telford, M.J., Glover, N.M., Dylus, D., *et al.*: OMA standalone: orthology inference among public and custom genomes and transcriptomes. Genome Res **29**(7), 1152–1163 (2019)
26. Pesquita, C., Faria, D., Bastos, H., Ferreira, A.E., Falcão, A.O., Couto, F.M.: Metrics for GO based protein semantic similarity: a systematic evaluation. BMC Bioinformatics **9**(Suppl 5), 4 (2008)
27. Friedland, S.: An upper bound for the number of perfect matchings in graphs. arXiv (0803.0864) (2008)
